# Polarity‐engineered Sn‐Ti cluster photoresists for sub‐10‐nm high‐resolution lithography

**DOI:** 10.1002/smo2.70061

**Published:** 2026-05-18

**Authors:** Daohan Wang, Runfeng Xu, Min Zhang, Xiaofeng Gong, Wenzheng Li, Danhong Zhou, Jun Zhao, Huie Zhu, Zhan Lu, Pengzhong Chen, Xiaojun Peng

**Affiliations:** ^1^ State Key Laboratory of Fine Chemicals Frontiers Science Center for Smart Materials Dalian University of Technology Dalian China; ^2^ Shanghai Synchrotron Radiation Facility Shanghai Advanced Research Institute Chinese Academy of Sciences Shanghai China; ^3^ Zhangjiang Laboratory Shanghai China; ^4^ College of Materials Science and Engineering Shenzhen University Shenzhen China

**Keywords:** lithography, molecular polarity, RLS trade‐off, sensitivity enhancement, Sn‐Ti cluster photoresist

## Abstract

High‐performance photoresists are essential for advancing semiconductor technology into the sub‐3‐nm process node. However, photoresist performance has long been constrained by the trade‐off among resolution (R), line edge roughness (L), and sensitivity (S), with the simultaneous enhancement of sensitivity and resolution remains a challenge. In this study, we propose a strategy to overcome the RLS trade‐off by enhancing the polarity of metal‐oxo clusters. A series of Sn‐Ti clusters with increasing polarity were synthesized by adjusting the bridging ligands and reducing the number of low‐polarity ligands. Experimental results confirm that, as the developer polarity decreases, higher polar Sn‐Ti clusters exhibit a more pronounced enhancement in sensitivity. The highest‐polarity cluster, TS‐3, undergoes a significant polarity switch upon exposure, enabling simultaneous improvement in sensitivity and resolution under lower‐polarity developers, ultimately breaking the conventional RLS trade‐off. TS‐3 delivers superior patterning performance in both electron beam lithography and extreme‐ultraviolet lithography, with a minimum linewidth of 8 nm. This study enhances the understanding of the solubility‐transition mechanism in cluster photoresists and provides a new approach for the development of high‐performance photoresists.

## Graphical abstract

Enhancing the polarity of clusters can optimize their patterning performance; inspired by this strategy, we designed polarity‐enhanced Sn‐Ti oxo clusters via ligand engineering. The optimal TS‐3 with exposure‐induced polarity switch achieves 8 nm patterning in lithography and achieves simultaneous improvement in resolution and patterning performance through developer polarity optimization. This research provides a new strategy for the development of advanced photoresists.

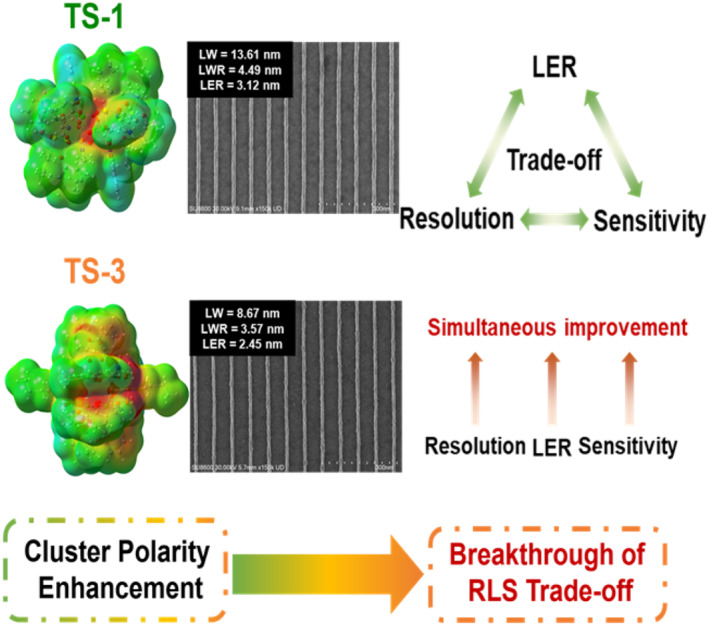

## 1 INTRODUCTION

The semiconductor industry continues to pursue ever‐shrinking feature sizes for fabricating higher‐performance and more energy‐efficient integrated circuits.^[1]^ According to the International Roadmap for Devices and Systems, the adoption of extreme‐ultraviolet (EUV) lithography systems with high numerical aperture by 2025 enabled semiconductor manufacturing at the sub‐3‐nm process node where the corresponding linewidth (LW) can reach 8 nm.^[2]^ This advancement brings the fundamental limitations of photoresist materials, particularly the longstanding trade‐off among resolution (R), line‐edge roughness (L), and sensitivity (S), commonly referred to as the “RLS trilemma.”^[3]^ Overcoming this trade‐off is crucial for high‐quality patterning at the sub‐3‐nm process node.

Currently, chemical amplification resists based on polymers, pioneered by IBM,[^4^, ^5^, ^6^] are intrinsically limited in high‐resolution lithography due to photoacid diffusion and its susceptibility to collapse during patterning.^[7]^ In response, researchers have proposed two solutions: molecular glass photoresists and organic–inorganic hybrid photoresists. Although molecular glass photoresists achieved improved patterning uniformity through refined particle sizes,[^3^, ^8^, ^9^, ^10^, ^11^, ^12^] particular attention has been focused on organic–inorganic hybrid systems, specifically metal cluster photoresists (e.g., Sn, Zn, Ti, Zr, and Hf‐based photoresists).[^13^, ^14^, ^15^, ^16^, ^17^, ^18^, ^19^, ^20^] These materials have emerged as highly promising candidates by uniquely combining high EUV absorption elements, small cluster sizes, and photoacid‐free patterning mechanisms.[^21^, ^22^, ^23^] Extensive research has focused on expanding the chemical diversity of cluster photoresists and elucidating their reaction mechanisms. However, few systems can deliver high‐quality patterning truly compatible with sub‐3‐nm process nodes, and strategies that can fundamentally overcome the RLS trade‐off remain scarce.

Thiols have been introduced into ZrO–MAA clusters to enhance sensitivity via the thiol‐ene click reaction while preserving patterning quality.^[24]^ Similarly, simultaneous enhancements were achieved in tin clusters by post‐exposure baking, which allowed for the continued reaction of species generated by low‐dose EUV.^[25]^ Further, pre‐exposing the Sn cluster photoresist film to deep ultraviolet prior to lithography improved sensitivity without compromising patterning quality. By increasing cluster polarity, this pre‐treatment reduces the dose required to induce a solubility transition during high‐energy exposure.^[26]^ Although the aforementioned strategies are effective, they increase the complexity and cost of the processes. Therefore, a more elegant solution would be to engineer the RLS performance intrinsically through molecular design, without the need for additives or extra processes.

Ti clusters have been regarded as a promising EUV lithography (EUVL) materials due to their high EUV absorption and excellent structural tunability.[^27^, ^28^] Zhang et al. improved the resolution to 12 nm while increasing sensitivity over 70% by simultaneously introducing thiols and double‐bond ligands into Ti clusters, without the additives or additional steps, highlighting the efficacy of intrinsic molecular engineering.^[29]^ Building upon this premise and inspired by the reported link between pre‐exposure and enhanced polarity, we hypothesized that systematic modulation of cluster polarity could offer a direct pathway to decouple the RLS trade‐off.^[26]^

In this study, we synthesized a series of Sn‐Ti clusters and successfully regulated the polarities of molecules by tailoring Sn‐Ti bridging ligands and adjusting the ratio of polar ligands to nonpolar ligands. We employed a molecular polarity index (MPI)‐based model to overcome the RLS trade‐off for the first time by regulating cluster and developer polarity, without additives or complex steps. Owing to the cluster polarity mutation after exposure, high‐polarity Sn‐Ti clusters TS‐3 enabled simultaneous enhancements in sensitivity and resolution as the developer's polarity decreases. Outstanding patterning results were achieved in e‐beam lithography (EBL) direct injection probe and EUVL processes, with LWs of 8.2 and 31.2 nm, respectively. This study provides a novel strategy to overcome critical performance bottlenecks, offering valuable guidance for designing future high‐performance photoresists.

## 2 RESULTS AND DISCUSSION

### 2.1 Clusters design and polarity calculation

The polarity and solubility of clusters are closely related to the properties of their surrounded ligands.[^15^, ^30^] Due to the exceptionally high EUV absorption cross‐section of Sn, Sn clusters have been widely explored in EUVL. During the formation of clusters, the highly polar Sn‐oxo core surrounded by low‐polarity alkyl chains, resulting in materials with overall low polarity.^[31]^ Current efforts to enhance the lithographic performance of Sn clusters have primarily focused on promoting Sn‐C bond cleavage and inter‐cluster cross‐linking interactions.[^32^, ^33^, ^34^, ^35^] Since patterning mechanism is fundamentally achieved through creating a solubility difference between exposed and unexposed regions in developer, the polarities of both the photoresist and the developer are critical aspects, yet underexplored.

**FIGURE 1:**
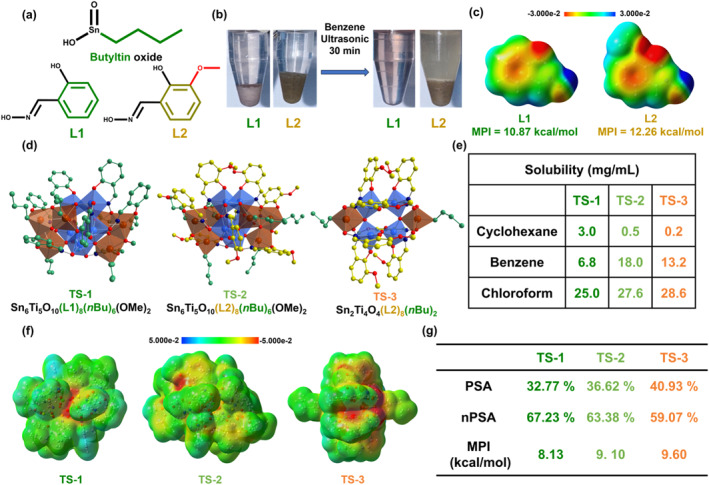
Structure and properties of ligands and Sn‐Ti clusters. (a) Chemical structures of butyltin oxide, 2‐hydroxybenzaldehyde oxime (L1), and 2‐hydroxy‐3‐methoxybenzaldehyde oxime (L2). (b) Differences in solubility of L1 and L2 ligands in benzene. (c) Their computed ESP contours and MPI. (d) Molecular structures of the Sn‐Ti composite clusters, TS‐1: Sn_6_Ti_5_O_10_(L1)_8_(*n*Bu)_6_(OMe)_2_, TS‐2: Sn_6_Ti_5_O_10_(L2)_8_(*n*Bu)_6_(OMe)_2_, and TS‐3: Sn_2_Ti_4_O_4_(L2)_8_(*n*Bu)_2_. Molecular properties of TS‐1, TS‐2, and TS‐3 obtained by experiments and theoretical calculations: (e) Solubility in cyclohexane, benzene, and chloroform, (f) ESP contours and (g) polar surface area, nonpolar surface area proportions and MPI value. ESP, electrostatic surface potentials; MPI, molecular polarity index.

To address this, constructing heterometallic Sn‐Ti clusters offers a promising strategy. The incorporation of Ti enriches structural diversity and provides a versatile platform to precisely modulate physicochemical properties. Herein, we designed a strategy to precisely tune cluster polarity via ligand engineering. We used a Ti‐oxo core bridged to Sn units capped with the ligands 2‐hydroxybenzaldehyde oxime (L1) and 2‐hydroxy‐3‐methoxybenzaldehyde oxime (L2). As shown in Figure 1a, the exposed parts of butyltin oxide and L1 are low‐polarity butyl chains and benzene rings, respectively. In contrast, ligand the introduction of a polar methoxy (CH_3_O) group in L2 is expected to increase its polarity relative to L1. The polarity difference was first confirmed experimentally by comparing their solubility in benzene, a low‐polarity solvent. The L1 ligand was completely dissolved while the L2 ligand exhibited poor solubility (Figure 1b), indicating its high polarity. For a quantitative comparison, the electrostatic surface potentials (ESP) of the two ligands were calculated (Figure 1c). Compared to the L1 ligand, the introduction of the CH_3_O group in L2 increased the nucleophilicity (red area). MPI is defined as the ratio of the integral of the absolute value of the molecular ESP to the molecular surface area, thus a higher MPI value indicates stronger molecular polarity. The MPI values were calculated to be 10.87 for L1 and 12.26 kcal/mol for L2, providing direct computational evidence for the designed polarity enhancement.^[36]^

Three Sn‐Ti composite clusters, viz., TS‐1 (Sn_6_Ti_5_O_10_(L1)_8_(*n*Bu)_6_(OMe)_2_), TS‐2 (Sn_6_Ti_5_O_10_(L2)_8_(*n*Bu)_6_(OMe)_2_), and TS‐3 (Sn_2_Ti_4_O_4_(L2)_8_(*n*Bu)_2_) were synthesized by adjusting the type of Sn‐Ti bridging ligands and the amount of organotin precursor (Figures 1d, S1–S4 and Tables S1–S3). TS‐1 and TS‐2 share the same core structure, with TS‐2 differed from TS‐1 by replacing the L1 ligand with the more polar L2 ligand. Although TS‐3 has a significantly different core structure from TS‐1 and TS‐2, its ligand types were the same as those of TS‐2. However, TS‐3 incorporates only two butyltin oxides, reducing the content of low‐polarity butyl chains compared to TS‐1 and TS‐2. Based on this molecular design, TS‐2 is expected to exhibit higher polarity than TS‐1 due to the high‐polarity of L2 ligands, while TS‐3 should possess higher polarity than TS‐2 owing to its lower proportion of nonpolar alkyl chains.

To validate this hypothesis, the solubility of the three clusters in cyclohexane, benzene, and chloroform were evaluated (Figures 1e and S5). In low‐polarity cyclohexane, TS‐1 exhibited relatively high solubility (∼3 mg/mL), whereas TS‐2 and TS‐3 were nearly insoluble. In benzene, the solubility of all three clusters improved, with TS‐2 and TS‐3 increasing 36‐fold and 66‐fold, respectively, far exceeding the 2.3‐fold increase for TS‐1, consistent with the higher polarity of TS‐2 and TS‐3.[^37^, ^38^] All three clusters exhibited good solubility in chloroform (25.0, 27.6, and 28.6 mg/mL for TS‐1, TS‐2, and TS‐3, respectively) due to the strong electrostatic interactions between clusters and chloroform.^[39]^ Therefore, chloroform was selected as the solvent for preparing the photoresist solution for subsequent tests.

Theoretical calculations further verified the polarity trend among the three clusters. From the ESP contours (Figure 1f), the ligand part of TS‐1 is electroneutral (green), and its nucleophilic part (red) is mainly confined to the O atoms within the metal‐oxo framework of the cluster, which are mostly shielded by the surrounding ligands. Compared with TS‐1, TS‐2 exhibits more exposed nucleophilic parts (yellow and red) on the surface because of the highly polar L2 ligands. This effect is further amplified in TS‐3, where only two butyltin units allow a greater ratio of highly polar L2 ligands and framework O atoms to be exposed, resulting in the highest proportion of nucleophilic regions among the series.

We define regions with |ESP| ≤ 10 kcal/mol as nonpolar surface area and |ESP| > 10 kcal/mol as polar surface area (PSA). Statistical analysis of the ESP distributions (Figure 1g) reveals that the PSA values gradually increase from TS‐1 (32.77%) to TS‐2 (36.62%) and further to TS‐3 (40.93%). The MPI calculation results are consistent with the above trend, which yield the order TS‐3 (9.60 kcal/mol) > TS‐2 (9.10 kcal/mol) > TS‐1 (8.13 kcal/mol). Previous studies have established that increasing cluster polarity prior to exposure can improve patterning performance.^[26]^ In this study, we demonstrate for the first time that such polarity enhancement can be achieved intrinsically through the regulating of the cluster structure itself, without relying on external additives or processing steps.

### 2.2 Cluster size, thermal stability, and film‐forming properties

**FIGURE 2:**
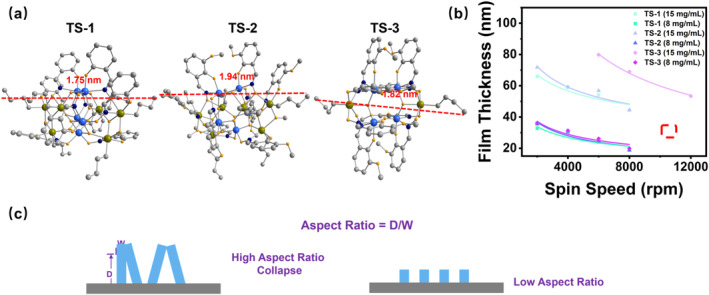
Cluster size and film‐forming properties of TS‐1, TS‐2, and TS‐3. (a) Cluster sizes measured by Diamond 4. (b) Film thickness curves of three clusters at different concentrations and spin speeds. (c) Calculation method of aspect ratio and schematic diagram of photoresist collapse.

To accurately determine the size of the three synthesized clusters, we to analyze their single crystal structures using Diamond 4 software (Figure 2a).^[40]^ The sizes for TS‐1, TS‐2, and TS‐3 are 1.75, 1.94, and 1.82 nm, respectively, which are‐suited for high‐resolution lithography applications.^[41]^ The thermal stability of the clusters, essential for withstanding the post‐application bake used to remove residual solvent after spin‐coating, was evaluated by thermogravimetric analysis (TGA). The test result shows that all clusters have a mass loss of 5% at relatively high temperatures: 283°C (TS‐1), 273°C (TS‐2), and 351°C (TS‐3) (Figure S6), indicating their sufficient thermal stability.

Thickness of photoresist films is another critical parameter affecting the lithography performance.^[42]^ The thicknesses of the films prepared at different concentrations and spin speeds were measured using atomic force microscopy (Figure 2b). While the film thickness gradually decreased with increasing spin speed, the concentration of 15 mg/mL yielded film thicker than 40 nm for all three clusters, even at a high spin speed. When the concentration was reduced to 8 mg/mL, the film thickness decreased to below 40 nm. Excessively thick films can result in a high aspect ratio and subsequent pattern collapse during the high‐resolution lithography process (Figure 2c).^[17]^ Therefore, industrial EUV processes typically require films thinner than ∼35 nm.^[43]^ In this study, the concentration and the spin speed were selected as 8 mg/mL and 4000 r/min, respectively, as the standard coating conditions (Figure 2b, red frame). Under these conditions, uniform films were obtained with a thickness of ∼30 nm and root mean square roughness of 0.39, 0.34, and 0.49 nm for TS‐1, TS‐2, and TS‐3, respectively (Figure S7). All three clusters achieved excellent surface flatness, providing a basis for high‐resolution lithography.^[44]^

### 2.3 Post‐exposure reaction and polarity change of Sn‐Ti composite clusters

To investigate the impact of cluster polarity on exposure performance, we used TGA‐mass spectrometry (TGA‐MS), direct injection probe‐mass spectrometry (DIP‐MS), and X‐ray photoelectron spectroscopy (XPS) to explore the exposure mechanism of the Sn‐Ti composite clusters.

**FIGURE 3:**
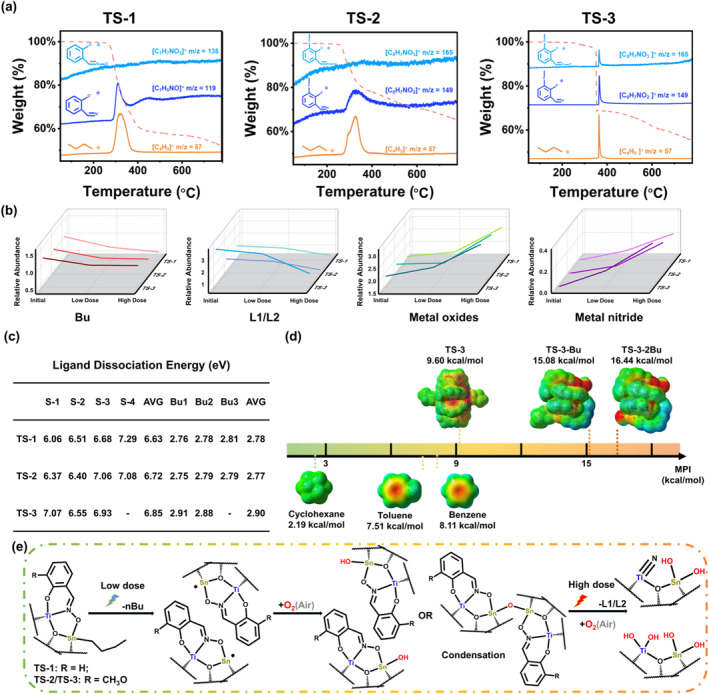
Reaction and polarity change in Sn‐Ti clusters after exposure. (a) TGA‐MS results. (b) Relative content of each component in the clusters after lithography obtained by X‐ray photoelectron spectroscopy test. (c) Calculated dissociation energies of L1/L2 and Bu ligands in three clusters under different chemical environments. (d) Molecular polarity index value of TS‐3 before and after exposure. (e) Reaction mechanism of Sn‐Ti clusters during exposure.

Analyzing the thermal decomposition behavior of the clusters can give preliminary insight into the changes they undergo upon exposure.^[45]^ The TGA‐MS results (Figure 3a) indicate that after reaching the cluster decomposition temperature, the organic ligands detached and generated abundant L1/L2 and butyl (Bu) fragments. No ligand fragments containing N‐O bonds (L1: [C_7_H_7_NO_2_]^+^ m/z = 135, L2: [C_8_H_7_NO_3_]^+^ m/z = 165) were detected in TS‐1 and TS‐2, while a small amount was detected in TS‐3. The L1/L2 ligands mainly dissociated from the clusters via the O‐M (M = Sn/Ti) bond and N‐O bond cleavage (L1: [C_7_H_5_NO]^+^ m/z = 119, L2: [C_8_H_7_NO]^+^ m/z = 149). Table S4 shows that the initial loss temperature of the Bu ligand is lower than that of the L1/L2 ligands, likely because the Sn‐C bond in the Bu ligand is weaker and easier to break.[^45^, ^46^] In addition, unlike L1/L2 ligands that bridge varying metal centers (Sn/Ti or Ti/Ti), the Bu ligand bonds solely to Sn, resulting in a single chemical environment and a correspondingly narrower dissociation temperature range.

In the EUVL and EBL, single photons or electrons carry high energy.^[47]^ To simulate the behavior of clusters under high‐energy beam exposure, we employ direct injection probe (DIP) transmission electron microscope technology to introduce clusters into a MS equipped with an Electron Ionization (EI) source. Directly bombarding the clusters with EI enables a more accurate simulation of high‐energy beam exposure processes and detection of the reaction products while eliminating solvent interference.^[48]^ DIP‐MS results (Table S5) showed that a large amount of Bu and L1/L2 ligand fragments were generated under the bombardment of high‐energy electrons, consistent with the TGA‐MS results. However, no L1/L2 ligand peak with N‐O bonds was found for all three clusters in DIP‐MS (Figures S8–S10). The above two MS results confirm that the organic ligands of the Sn‐Ti cluster detach after stimulation. For butyltin oxide, Sn‐C bond cleavage primarily causes Bu detachment, followed by further alkyl chain breakage. The detachment of L1/L2 ligands is mainly caused by O‐M and N‐O bond cleavage.

XPS was performed to analyze the composition in the photoresist films before and after low‐dose (100 μC/cm^2^) and high‐dose (600 μC/cm^2^) EBL tests to further explore the changes in the two types of ligands during the exposure process (Figure S11). C 1s spectra were deconvoluted into three components: 285.7 eV for C‐O/C=N, 284.4 eV for C‐C/C‐H/C=C, and 283.9 eV for Sn‐C.[^49^, ^50^, ^51^] At both exposure doses, the contents of Sn‐C and C‐O/C=N for the three clusters decreased after exposure, indicating the loss of Bu and L1/L2 ligands. This loss, together with cross‐linking among the generated alkyl radicals leads to an increase in the C‐C component. O 1s spectra were deconvoluted into three components: 532.5 eV for CH_3_‐O (TS‐2 and TS‐3), 531.4 eV for aromatic‐O (Ar‐O)/N‐O, and 530.0 eV for metal oxides.^[52]^ After exposure, CH_3_‐O and Ar‐O/N‐O decreased due to the loss of L1/L2 ligands. Concurrently, metal active sites were exposed after ligand desorption and further reacted with atmospheric O_2_, leading to an increased metal oxide contents.^[32]^ N 1s spectra before exposure were deconvoluted into two components: 402.9 eV for π‐π* satellite peak, and 399.8 eV for C=N‐O.[^53^, ^54^] The ratios of both chemical components decreased upon exposure due to the loss of L1/L2 ligands. Interestingly, a new peak appeared at 398.8 eV after exposure, which is related to metal nitride^[55]^ and became more obvious at high exposure doses.

The Sn content remained constant before and after exposure. Accordingly, the contents of Sn‐C, C=N‐O, metal oxides and metal nitrides determined by XPS were normalized to the Sn content, to obtain the relative contents of Bu, L1/L2, metal oxides and metal nitrides in the thin films at different exposure doses. The variation profiles of these relative contents as a function of exposure dose were subsequently plotted in Figure 3b. At low exposure doses, the Bu ligand content decreased sharply but then plateaued. In contrast, the L1/L2 ligands exhibited minor losses at low doses, with significant ligand detachment at higher doses. At the same time, the increase in metal oxide content was not obvious at a low exposure dose, likely because only one Sn was exposed after the removal of Bu ligands, whereas the dissociation of the L1/L2 ligand at high doses liberated more active metal sites (Ti or Sn). Thus, the metal oxide contents were significantly increased after high‐dose exposure. Similarly, the metal nitride content increased significantly only after extensive dissociation of the L1/L2 ligands at high dose. All these observations demonstrate that the Sn‐C bond having lower bond dissociation energy, dissociate more readily at lower exposure doses, compared to L1/L2 ligands, consistent with the TGA‐MS results.

Theoretical calculations further validated these observations. Because of the distinct chemical environments of the ligands, we divided the L1/L2 ligands of TS‐1/TS‐2 into four groups and the Bu ligands into three groups. For TS‐3, the ligands were classified into three L2 and two Bu groups (Figure S12). The dissociation energies of the two types of ligands in their respective chemical environments were calculated.

As shown in Figure 3c, the dissociation energies of the L1/L2 ligands in TS‐1, TS‐2, and TS‐3 are 2.38, 2.43, and 2.36 times higher than those of the Bu ligands, respectively. In addition, the chemical environments of the L1/L2 ligands differed significantly, as reflected in the difference between the highest and lowest dissociation energies: 1.23 eV (TS‐1), 0.71 eV (TS‐2), and 0.52 eV (TS‐3). These ranges are substantially wider than those of the butyl ligand: 0.05 eV (TS‐1), 0.04 eV (TS‐2), and 0.03 eV (TS‐3). The calculated bond dissociation energies confirmed that Bu, with their lower and narrower distributed bond energies, dissociated more readily and nearly simultaneously upon exposure, when compared with L1/L2 ligands.

Based on the above tests, changes in the MPI values of the clusters before and after exposure were calculated using TS‐3 as an example (Figure 3d). The MPI of TS‐3 before exposure was 9.60 kcal/mol, and those of cyclohexane, toluene, and benzene were 2.19, 7.51, and 8.11 kcal/mol, respectively. According to the principle of “like dissolves like,”^[56]^ the smaller the difference between the MPI values of the solute and solvent, the better the solubility, which explains why TS‐3 dissolves better in benzene during solubility testing.^[29]^ Due to the low content of nonpolar Bu ligands, TS‐3 exhibited a significant polarity switch after exposure. The MPI values of TS‐3 after the loss of one and two Bu ligands were 15.08 and 16.44 kcal/mol, respectively. Such high MPI values render the exposed clusters insoluble in the above three solvents, where were therefore selected as developers. We believe that this sharp polarity switch in TS‐3, triggered by losing only a small number of Bu ligands, is key to the improvement of the cluster lithography performance.

The exposure mechanism of the Sn‐Ti clusters under high‐energy beams is proposed (Figure 3e), based on the combined experimental and theoretical evidence. At low exposure doses, the weaker Sn‐C bonds break first, forming alkyl radicals and active Sn sites. The ambient O_2_/H_2_O subsequently reacted with the exposed Sn sites to produce tin oxides, increasing the cluster polarity.[^33^, ^57^, ^58^, ^59^] Further increasing the exposure dose caused the dissociation of the L1/L2 ligands, thereby exposing more active metal sites (Sn/Ti). Their subsequent oxidation further elevated the metal‐oxide content and overall polarity. The amplified polarity difference between exposed and unexposed areas resulted in sufficient solubility contrast in the developer, enabling high‐fidelity pattern transfer.^[26]^

### 2.4 Optimizing exposure performance by adjusting developer polarity

The lithographic performance of the three clusters was optimized using the line mode of the EBL device. In this mode, the electron beam is exposed only once along the drawn path to obtain high‐resolution patterns.

**FIGURE 4:**
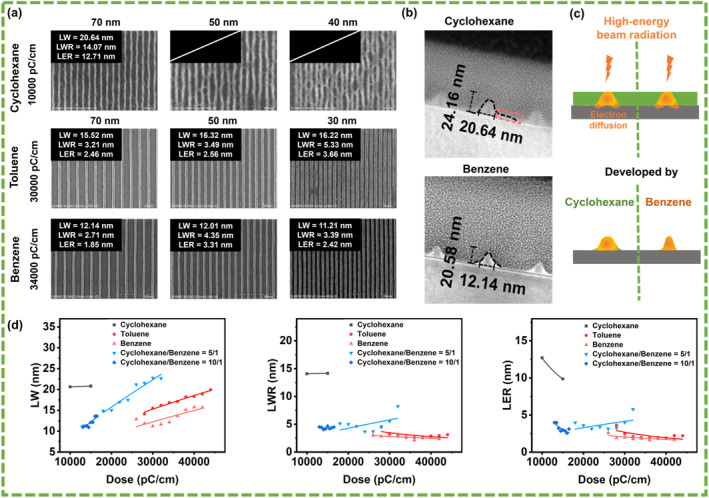
Optimization of developer formulation to improve the exposure performance of TS‐1. (a) Lines obtained under different pitches and developers. (b) Cross‐sectional image of lines under different solvents, obtained by FIB‐TEM. (c) Schematic of development variations. (d) Line graphs of linewidth, line width roughness, and LER as a function of exposure dose using different developers.

TS‐1 was developed using three solvents with increasing polarity: cyclohexane, toluene, and benzene. As shown in Figure 4a, TS‐1 exhibited the highest sensitivity with the least polar developer cyclohexane, and achieving patterning at 10,000 pC/cm. The resolution and quality of the pattern were poor, with a minimum LW of 20.64 nm, and the pattern collapsed when the pitch was reduced to 50 nm. The use of higher‐polarity toluene and benzene significantly improved the patterning performance, allowing a minimum pitch of 30 nm. Toluene achieved a minimum LW of 15.52 nm, while benzene further yielded an even smaller LW of 11.21 nm. However, this improvement in resolution came at the cost of sensitivity, as the required exposure dose increased to 30,000 and 34,000 pc/cm, respectively.

Cross‐sections of the lines developed with cyclohexane and benzene were observed by focused ion beam‐transmission electron microscope (FIB‐TEM, Figure 4b). Compared to benzene, cyclohexane development resulted in broader and thicker lines. In addition, bridging occurred in the unexposed regions between the lines (red‐framed areas). When benzene was used as the developer, the non‐exposed areas were completely dissolved, yielding a sharper line boundary. This difference may be attributed to the electron scattering and the secondary‐electron diffusion during exposure, which caused partial ligand loss in areas adjacent to the exposed regions (Figure 4c).[^42^, ^60^, ^61^] The low‐polarity cyclohexane cannot be completely dissolved these partially affected clusters, leading to LW increasing or bridging. In contrast, highly polar benzene can effectively dissolve the partially exposed clusters, producing cleaner developmental outcomes.

To improve the sensitivity without sacrificing pattern quality, cyclohexane was mixed with benzene for development (Figure S13). As the volume ratio of cyclohexane increased, the required exposure dose gradually decreased. When cyclohexane/benzene = 10/1, the dose dropped to 14,500 pC/cm, approaching that of pure cyclohexane. The improvement in sensitivity did not compromise patterning performance; the minimum LW remained as low as 10.91 nm. We measured the lines obtained by different developers at a pitch of 70 nm (Figures S14–S18), and plotted the curves of LW, line edge roughness (LER), and line width roughness (LWR) as a function of the exposure dose (Figure 4d). The line graph demonstrates that benzene and toluene have little effect on the sensitivity, LWR, and LER for TS‐1, but benzene a can achieve a smaller LW. Although a mixture of cyclohexane and benzene can enhance the exposure sensitivity of TS‐1 and achieve high‐resolution lines at low doses, this improvement diminishes as the exposure dose increases, indicating a narrow process window. This phenomenon may arise because of the low‐polarity L1 ligand in TS‐1. Small numbers of Bu detachment cause negligible polarity changes for the solubility shift. However, increased exposure doses promote electron diffusion and extend the partially exposed areas.

**FIGURE 5:**
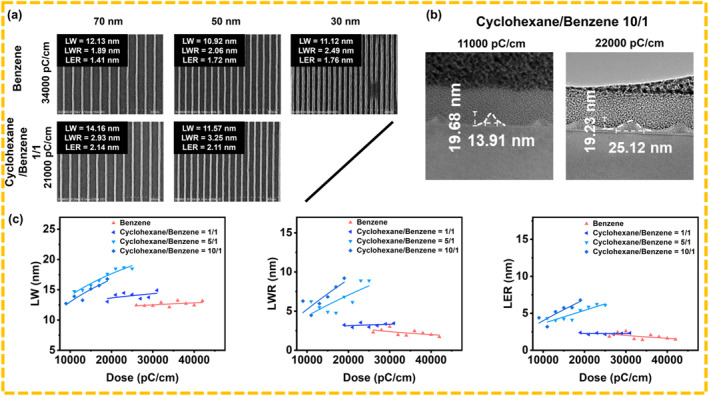
Optimization of developer formulation to improve the exposure performance of TS‐2. (a) Pattern performance using different developers. (b) Cross‐sectional image of lines at different doses. (c) Line graph of patterning performance as a function of exposure dose using different developers.

The performance of TS‐2 is similar to that of TS‐1. When developed with benzene, TS‐2 required the same dose as TS‐1 (34,000 pC/cm) and achieved a similar minimum LW (10.92 nm) and pitch (30 nm) (Figure 5a). With the increase of cyclohexane volume in the developer, TS‐2 exhibited a more pronounced sensitivity improvement than TS‐1. At the cyclohexane/benzene ratio of 10/1, only 9000 pC/cm was required for patterning, lower than the dose needed for TS‐1 developed in pure cyclohexane (Figure S19). However, cross‐sectional images obtained via FIB‐TEM revealed that increased exposure doses still caused line broadening when a low‐polarity developer was used (Figure 5b), leaving a thin residue in unexposed regions. The line graphs for TS‐2 patterns fabricated at a 70 nm pitch in different developers are shown in Figures 5c and S20–S23. Compared to TS‐1, the sensitivity of TS‐2 increased more obviously when the developer polarity was reduced, but a trend of LW enlargement was still observed. Furthermore, the LWR and LER curves showed that the line quality decreased significantly as the developer polarity decreased. These results indicate that the high polarity L2 ligand in TS‐2 promotes a more abrupt solubility transition in the low‐polarity developer after exposure. However, owing to the high content of the Bu ligand, a certain amount of Bu detachment is still required to trigger the change in solubility, and electron diffusion effects during this process likely contribute to the observed decline in pattern quality.

**FIGURE 6:**
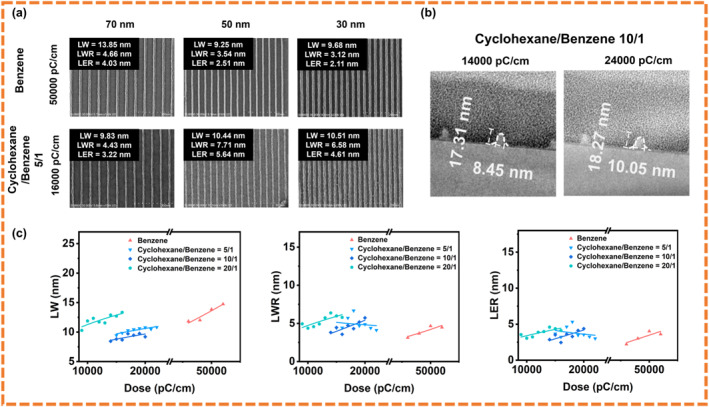
Optimization of TS‐3 patterning performance by modulating the developer polarity. (a) Patterning results of TS‐3 under different developers and pitches. (b) Cross‐sectional images of lines at different doses when the developer was cyclohexane/benzene = 10/1. (c) Line graphs of linewidth, line width roughness, and LER as a function of exposure dose using different developers.

Among the three clusters, TS‐3 exhibited the lowest sensitivity (50,000 pC/cm) when benzene was used as the developer, likely due to its average higher bond dissociation energy. Nevertheless, TS‐3 demonstrated an exceptionally high resolution, achieving a minimum LW of 9.25 nm (Figure 6a), consistent with the RLS trade‐off.[^3^, ^62^, ^63^] Notably, the addition of cyclohexane to the developer significantly enhanced the sensitivity, enabling patterning at 10,000 pc/cm. This increase in sensitivity did not compromise patterning performance. Instead, the minimum LW was further reduced to 8.21 nm. Furthermore, the LER and LWR of the lines remained largely unchanged (Figure S24). The cross‐section of the lines obtained by the low‐polarity developer showed that an increase in the exposure dose did not lead to LW broadening or residual scum in the non‐exposed area (Figure 6b).

The line graph directly illustrated how the exposure performance of TS‐3 changed with the developer (Figures 6c and S25–S28). Since TS‐3 possesses highly polar L2 ligands and only two Bu ligands, the detachment of even a small number of Bu ligands during exposure can significantly alter the cluster polarity. Therefore, reducing the developer's polarity markedly enhancing TS‐3's sensitivity. At the same time, the high dissociation energy of TS‐3 makes the non‐exposed area less susceptible to electron‐scattering effects. Thus, even using low‐polarity developers, its LW, LWR, and LER are minimally affected by the increased exposure dose, ensuring a wide process window. Under these conditions, a lower dose can achieve smaller features while maintaining excellent LER and LWR performance.

**FIGURE 7:**
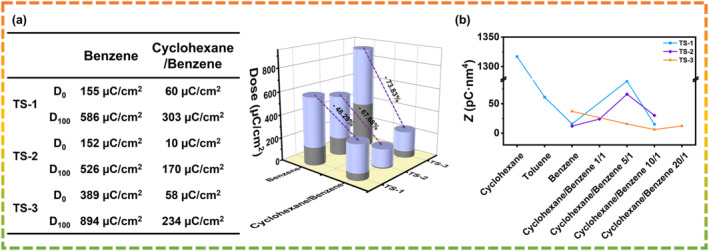
Comparison of pattern performance improvements for TS‐1, TS‐2, and TS‐3 before and after developer optimization: (a) D_0_ and D_100_ of clusters before and after reducing the developer's polarity. (b) Line graph of minimum *Z*‐values for patterned lines using different developers.

By adjusting the polarity of the developer, we successfully enhanced the sensitivity of the three clusters, while maintaining optimal development performance. Dose array tests revealed that the D_100_ (the lowest dose for completely insolubility) decreased from 586, 526, and 894 μC/cm^2^ to 303, 170, and 234 μC/cm^2^ after optimization, for TS‐1, TS‐2, and TS‐3, respectively (Figures S29–S31). Clusters with higher polarity are more sensitive to reductions in developer polarity. The sensitivity of the highest‐polarity TS‐3 increased by 73.83%, followed by lower‐polarity TS‐2 (67.68%) and the lowest‐polarity TS‐1 (48.29%) (Figure 7a).

A *Z*‐value was introduced to provide a more intuitive comparison of the lithographic performance. The *Z*‐value is defined as *Z* = *R* ^3^ × *L* ^2^ × *D*, where *R* is the LW (nm), *L* is the LER (nm), and *D* is the dose or sensitivity (pC/cm).A smaller *Z*‐value indicates a better performance of the photoresist.[^60^, ^64^] We calculated the *Z*‐values for the three clusters using different developers (Figures S32–S34) and plotted a line graph using the lowest *Z*‐value for each condition (Figure 7b). TS‐1 developed with low‐polarity cyclohexane achieved high sensitivity but poor patterning performance, with *Z*‐values reaching 1316.87 pC·nm^4^. Using highly polar developers, such as toluene and benzene, reduced the LW and LER of TS‐1 at the expense of sensitivity, resulting in a significant reduction in *Z*‐value to 15.81 pC·nm^4^. Although blending developers with different polarities could enhance sensitivity, for the less polar TS‐1 and TS‐2, LW and LER were adversely affected, resulting in no improvement in *Z*‐value. For TS‐3 with higher polarity, owing to its sharp polarity switch and higher ligand dissociation energy, the blended developer not only improved its sensitivity, but also achieved smaller LW and LER at lower doses. Using cyclohexane/benzene = 10/1 as the developer yielded a minimum *Z*‐value of 6.26 pC·nm^4^, which was only half that of TS‐1 and TS‐2 under their optimal conditions.

**FIGURE 8:**
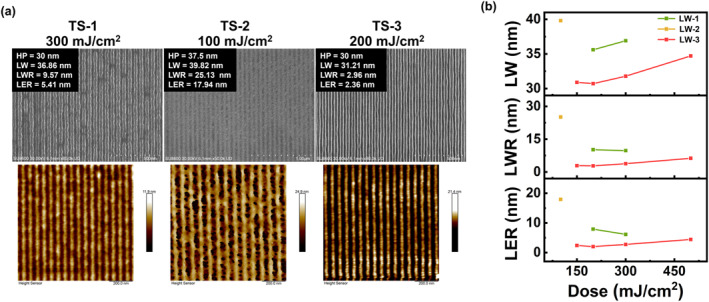
EUVL test results of TS‐1, TS‐2, and TS‐3 using cyclohexane/benzene = 10/1 as a developer. (a) SEM and atomic force microscopy images of the lines obtained by EUVL. (b) Line graph of EUVL test results for three clusters.

Because TS‐1, TS‐2, and TS‐3 all achieved low *Z*‐values with a developer composition of cyclohexane/benzene = 10/1, we selected this formulation as the developer for EUVL testing. The EUV patterning results were consistent with the trends observed in EBL. Under identical developer conditions, TS‐2 demonstrated high sensitivity (Figure 8a), requiring only 100 mJ/cm^2^ to form a pattern; however, its patterning fidelity was poor. At half pitch of 37.5 nm, LW, LWR, and LER reached 39.82, 25.13, and 17.94 nm, respectively. The LW expansion was evident as the exposure dose increased (Figure S35). The lowest‐polarity TS‐1 requires the highest exposure dose (200 mJ/cm^2^ under EUV irradiation) to pattern. Although the patterning result of TS‐1 showed some improvement, its LWR and LER were still poor due to the noticeable line blur and residue in unexposed regions induced by high exposure dose. TS‐3, which had the highest polarity, not only showed high sensitivity (patterning at 150 mJ/cm^2^), but also ensured pattern fidelity. At a dose of 200 mJ/cm^2^, its LW reached 31.21 nm with LWR and LER of only 2.96 and 2.63 nm, respectively, significantly outperforming both TS‐1 and TS‐2. Plotting all data as a line graph reveals that the increase in the polarity of the Sn‐Ti clusters also enhances their overall EUVL performance (Figure 8b). As the developer polarity decreased, the sensitivity of the high‐polarity Sn‐Ti clusters increased. Through structural optimization, the highest‐polarity TS‐3 achieved simultaneous improvement in sensitivity and resolution, overcoming the RLS trade‐off. Furthermore, TS‐3 exhibited a broad process window, maintaining good patterning performance even at a dose as high as 500 mJ/cm^2^.

### 2.5 Etch resistance

To achieve high‐resolution patterning, photoresist films must combine minimal thickness with high etch resistance.^[3]^ The etch resistance of the three Sn‐Ti composite clusters was evaluated by an inductively coupled plasma method using SF_6_ as the etching gas (Table S6). After etching for 5 s, the thicknesses of TS‐1, TS‐2, and TS‐3 increased by 9.58, 10.39, and 17.11 nm, respectively, yielding etching rates of 5.08, 4.92, and 3.58 nm/s (Figure S36). TS‐3 exhibited a higher selection ratio of 1.95/1 relative to Si (7 nm/s), compared with TS‐1 and TS‐2, which may be attributed to its higher Ti content. All three clusters demonstrated remarkable etch resistance, making them suitable for high‐resolution pattern transfer.

## 3 CONCLUSION

In this study, we engineered the polarity of the Sn‐Ti composite cluster and developers for nanopatterning. For the first time, the conventional RLS trade‐off was overcome by regulating the cluster polarity without involving additives or complex processes. Combined with the decrease of developer polarity, the high‐polarity TS‐3 exhibited a 1.04 nm LW reduction along with a 73.83% increase of sensitivity. It achieved high‐quality patterns in both EBL and EUVL, with LWs of 8.2 and 31.2 nm, respectively. Theoretical calculations indicated that clusters with fewer low‐polarity alkyl ligands undergo polarity mutation more readily during exposure, leading to improved lithographic performance. This study provides valuable insights into the reaction mechanisms of high‐energy irradiation photoresists and establishes a novel approach for overcoming the RLS trade‐off in the development of high‐resolution cluster photoresists.

## CONFLICT OF INTEREST STATEMENT

The authors declare no conflicts of interest.

## ETHICS STATEMENT

No animal or human experiments were involved in this study.

## Supporting information

Supporting Information S1

Supporting Information S2

## Data Availability

CCDC: 2471499 (TS‐1), 2471496 (TS‐2), and 2471492 (TS‐3) contains the supplementary crystallographic data for this paper. These data can be obtained free of charge from The Cambridge Crystallographic Data Centre via www.ccdc.cam.ac.uk/data_request/cif.
