# Supplementary material for: Polarity‐engineered Sn‐Ti cluster photoresists for sub‐10‐nm high‐resolution lithography
Source: Smart Mol. 2026 May 18:e70061. Online ahead of print. doi: 10.1002/smo2.70061 (PMC13399119; doi:10.1002/smo2.70061)
Supplement: Supplementary file 1 — Supporting Information S1 [file SMO2-9999-0-s001.docx]

*Supporting Information*

**Polarity-Engineered Sn–Ti Cluster Photoresists for Sub-10-nm High-Resolution Lithography**

Daohan Wang^1^, Runfeng Xu^1^, Min Zhang^1^, Xiaofeng Gong^1^, Wenzheng Li^1^, Danhong Zhou^1^, Jun Zhao^2^, Huie Zhu^3^, Zhan Lu^3^, Pengzhong Chen^1, *^, Xiaojun Peng^1, 4, *^

^1^State Key Laboratory of Fine Chemicals, Frontiers Science Center for Smart Materials, Dalian University of Technology, Dalian 116024, P. R. China

^2^Shanghai Synchrotron Radiation Facility, Shanghai Advanced Research Institute, Chinese Academy of Sciences, Shanghai 201204, P. R. China

^3^Zhangjiang Laboratory, 100 Haike Road, Pudong New Area, Shanghai 201210, P. R. China

^4^College of Materials Science and Engineering, Shenzhen University, Shenzhen 518060, P. R. China

* E-mail: pengxj@dlut.edu.cn; pzchen@dlut.edu.cn

Contents

[1. General information 3](#_Toc225873148)

[2. Synthesis and Testing Procedure 5](#_Toc225873149)

[2.1 Materials Preparation and Characterization 5](#_Toc225873150)

[2.2 X-ray Crystallography 7](#_Toc225873151)

[2.3 Cluster size Distribution 7](#_Toc225873152)

[2.4 Film Deposition 8](#_Toc225873153)

[2.5 Lithography performance test and optimization 8](#_Toc225873154)

[2.6 Acquisition of patterned line cross section 9](#_Toc225873155)

[2.7 Theoretical Calculations: 10](#_Toc225873156)

[3. Supplementary Content 12](#_Toc225873157)

[4. References 44](#_Toc225873158)

# General information

In this report, reagents Titanium propoxide and Butyltin oxide were purchased from Aldrich, reagents 2-Hydroxybenzaldehyde oxime (L1) and 2-Hydroxy-3-methoxybenzaldehyde oxime (L2) from Bide Pharmatech Co. Ltd, Formic acid, Propionic acid, and extra dry Methanol (MeOH) from Energy Chemical. All reagents were used as received.

NMR spectra were detected by Bruker Avance III 400 spectrometer. Chemical shift (*δ*) was reported as ppm in Methylene Chloride-*d*_2_ (TS-1) or DMSO-*d*_6_ (TS-2 and TS-3) with TMS as the internal standard. Elemental analysis was performed by Elementar's UNICUBE/OXYCUBE. Crystallographic data were collected on XtaLAB Synergy Custom which was equipped with single source at home/near Mo X-ray sources (λ = 0.71073 Å) at 100 K. Transmission electron microscopy (TEM) images were performed on JEM-F200 (JEOL Ltd.). Thermal gravimetric analysis (TGA) measurements were performed using a TGA/DSC 3+ instrument. Sample was heated from 50℃ to 800℃ at a rate of 10°C/min under an argon atmosphere, and the extent of thermal weight loss was recorded. Mass spectrometry testing using Orbitrap Exploris GC equipped with Direct Probe (DIP-MS). X-ray Photoelectron Spectroscopy (XPS) were performed on Thermo Fisher Scientific ESCALAB Xi+. Atomic force microscope (AFM) images were performed on Bruker’s Dimension Icon. Electron beam lithography (EBL) testing is conducted by the Hitachi Scanning Electron Microscope (SEM) SU8600 equipped with Raith ELPHY Quantum Nanofabrication systems. Hitachi SEM SU8600 and Bruker Dimension Icon AFM is used for image inspection after E-beam Lithography (EBL) and Extreme Ultraviolet Lithography (EUVL). The cross-sections of the exposed lines were prepared using TESCAN SOLARIS FIB-SEM system with a gallium ion source, and the sample were observed using JEM-F200 (JEOL Ltd.).

# Synthesis and Testing Procedure

## Materials Preparation and Characterization

**Sn_6_Ti_5_O_10_(L1)_8_(*n*Bu)_6_(OMe)_2_ (TS-1):** 2-Hydroxybenzaldehyde oxime (L1) (137 mg, 1 mmol) and Butyltin oxide (150 mg, 0.72 mmol) was fully dissolved in 3 mL MeOH. Add 100 μL Titanium propoxide and 100 μL Propionic acid to the above solution. The resulting mixture was sealed and subjected to ultrasonic treatment for 30 minutes to ensure thorough mixing, and then placed in a constant temperature oven at 90℃ for 4 d. Light yellow crystal were produced at the bottom, product was filtered off, washed with MeOH and dried at 40°C in vacuum 24 hours, yield: 143 mg (46% rel. to Sn)^[1]^.

^1^H NMR (400 MHz, Methylene Chloride-*d*_2_, ppm): 8.31-7.63 (m, 8H), 7.34-7.22 (m, 8H), 7.08-6.90 (m, 8H), 6.86-6.72 (m, 8H), 6.68-6.37 (m, 8H), 4.02-3.65 (m, 6H), 1.84-1.09 (m, 36H), 1.02-0.50 (m, 18H).

Elemental analysis: Anal. Calcd for Sn_6_Ti_5_C_82_H_100_O_28_N_8_ (weight %): C, 37.92; H, 3.88; O, 17.24; N, 4.31; Ti, 9.21; Sn, 27.41. Found: C, 39.25; H, 3.69; N, 4.46.

**Sn_6_Ti_5_O_10_(L2)_8_(*n*Bu)_6_(OMe)_2_ (TS-2):** 2-Hydroxy-3-methoxybenzaldehyde oxime (L2) (167 mg, 1 mmol) and Butyltin oxide (150 mg, 0.72 mmol) fully dissolved in 3 mL MeOH. Add 100 μL Titanium propoxide and 100 μL Formic acid to the above solution. After ultrasonic dissolution for 30 min, the mixture was sealed and transferred to a constant temperature oven at 90℃ for 5 days. Orange crystal were produced at the bottom, product was filtered off, washed with MeOH and dried at 40℃ in vacuum 24 hours, yield: 119 mg (35% rel. to Sn)^[1]^.

^1^H NMR (400 MHz, DMSO-*d*_6_, ppm): 8.31-7.57 (m, 8H), 7.24-6.91 (m, 8H), 6.83-6.41 (m, 8H), 6.39-6.02 (m, 8H), 4.33-3.50 (m, 24H), 3.32 (d, *J* = 6.7 Hz, 6H), 2.09-1.13 (m, 30H), 0.97 (t, *J* = 7.4 Hz, 6H), 0.86 (t, *J* = 7.3 Hz, 6H), 0.69-0.45 (m, 6H), 0.30 (t, *J* = 7.2 Hz, 6H).

Elemental analysis: Anal. Calcd for Sn_6_Ti_5_C_90_H_116_O_36_N_8_ (weight %): C, 38.09; H, 4.12; O, 20.29; N, 3.95; Ti, 8.43; Sn, 25.11. Found: C, 39.23; H, 4.03; N, 3.72.

**Sn_2_Ti_4_O_4_(L2)_8_(*n*Bu)_2_ (TS-3):** 2-Hydroxy-3-methoxybenzaldehyde oxime (L2) (167 mg, 1 mmol) and Butyltin oxide (150 mg, 0.72 mmol) fully dissolved in 3 mL MeOH. Add 100 μL Titanium propoxide and 100 μL Propionic acid to the above solution. After ultrasonic dissolution for 30 min, the mixture was sealed and transferred to a constant temperature oven at 90℃ for 5 days. Orange crystal were produced at the bottom, product was filtered off, washed with MeOH and dried at 40℃ in vacuum 24 hours, yield: 91 mg (13% rel. to Sn).

^1^H NMR (400 MHz, DMSO-*d*_6_, ppm):7.67 (s, 8H), 6.70 (d, *J* = 8.0 Hz, 8H), 6.44-6.35 (m, 8H), 6.23-5.92 (m, 8H), 4.20-3.70 (m, 24H), 1.76 (s, 4H), 1.46 (s, 4H), 1.13 (s, 4H), 0.99-0.93 (m, 6H).

Elemental analysis: Anal. Calcd for Sn_2_Ti_4_C_72_H_74_N_8_O_28_ (weight %): C, 44.85; H,3.87; O, 23.23; N,5.81; Ti, 9.93; Sn, 12.31. Found: C, 45.67; H, 3.69; N, 6.02.

## X-ray Crystallography

Crystallographic data of these structures were collected on XtaLAB Synergy Custom which was equipped with single source at home/near Mo X-ray sources (λ = 0.71073 Å) at 100 K. The structures were solved with dual-direct methods using ***SHELXTL*** and refined with the full-matrix least-squares technique based on *F^2^* using the ***SHELXL*-2014** program package and ***Olex-2*** software.^[2]^ Non-hydrogen atoms were refined anisotropically, and all hydrogen atoms bound to C were generated geometrically. The crystal data of these compounds are listed in Table S1. The X-ray crystallographic coordinates for structures reported in this article have been deposited at the Cambridge Crystallographic Data Centre (CCDC) under deposition numbers CCDC: 2471499 (TS-1), 2471496 (TS-2) and 2471492 (TS-3). These data can be obtained free of charge from the Cambridge Crystallographic Data Centre via <http://www.ccdc.cam.ac.uk/data_request/cif>. ***Diamond 4.6.8*** is used to draw the parsed structure.

## Cluster size Distribution

Use a capillary tube to dip a small amount of the product into anhydrous ethanol to make it dissolve and 2 µL of the liquid was dropped on a 300 mesh Ultrathin Pure Carbon Film with no Formvar Backing on Lacey Carbon Support Film. The samples were photographed by TEM and the cluster size were measurement and statistics by **Nano Measurer 1.2**. The theoretical size of the cluster is measured by **Diamond 4.6.8**.

## Film Deposition

Thin films of **TS-1**, **TS-2**, and **TS-3** were prepared by spin-coating a solution from chloroform at different concentrations and spin speed. After coating, Post-Apply Bake (PAB) was carried out at 60°C for 60 s using a hot plate, so that the solvent in the photoresist was completely volatilized. The morphology and thickness of the film was measured by AFM.

## Lithography performance test and optimization

**EBL:**

Exposure of the dosage array was performed using area exposure mode with a 50 μm APT size, 2 kV accelerating voltage, 10 μA probe current, and 1 spot size. The data with normalized thickness between 0.1 - 0.9 are fitted, and the intersection points of the obtained tangent and the straight lines with normalized thickness of 0 and 1 are recorded as D_0_ and D_100_, respectively.

Exposure of the lines was performed in line exposure mode with a 30 μm APT size, 30 kV accelerating voltage, 1 μA probe current, and 1 spot size.

All samples were directly developed after exposure. TS-1 was developed with Cyclohexane (40 s), Toluene (20 s), Benzene (20 s), Cyclohexane/Benzene = 5/1 (30 s), and Cyclohexane/Benzene = 10/1 (40 s), respectively. TS-2 was developed with Benzene (20 s), Cyclohexane/Benzene = 1/1 (30 s), Cyclohexane/Benzene = 5/1 (30 s), and Cyclohexane/Benzene = 10/1 (40 s), respectively. TS-3 was developed with Benzene (20 s), Cyclohexane/Benzene = 5/1 (30 s), Cyclohexane/Benzene = 10/1 (40 s), and Cyclohexane/Benzene = 20/1 (40 s), respectively. All samples were dried by a nitrogen gun after development.

**EUVL:**

EUVL carried out at soft X-ray interference lithography beamline (BL08U1B) in Shanghai Synchrotron Radiation Facility (SSRF). Dense lines are obtained by double-beam interference technique. After exposure all samples were directly developed with Cyclohexane/Benzene = 10/1 (40 s) and dried by a nitrogen gun after development.

## Acquisition of patterned line cross section

Using the TESCAN SOLARIS FIB-SEM system, the cutting site was first precisely located under an electron beam, the cutting area was coated with Pt for protection using the electron beam, and then the Pt protective layer is deposited again under the ion beam. The sample was taken out and welded on the copper mesh and thinned using a focused ion beam and a nano-manipulator. The obtained samples were photographed under TEM.

## Theoretical Calculations:

The metal-oxo cluster structures used for the calculations were adopted the results of single crystal structure. Because of the extreme speed during high-energy excitation, the parent structure is not changed in time during ionization and ligand dissociation, but the ligand undergoes rapid structural relaxation in the high-energy state. Therefore, only the geometric structure of the dissociated ligand is optimized for all computations, whereas the parent and the parent residue with one ligand removed are not structurally optimized. The optimization of the geometry of the ligand and the calculation of the single-point energies of the individual structures were carried out using DFT, choosing the PBE0 functional, and selecting the def2svp basis set for all atoms, which already contains the pseudopotentials for the fourth-period Ti and Sn elements. The ligand dissociation energy of the cluster is obtained by subtracting the single-point energy of the intact cluster before dissociation from the sum of the single-point energies of the residual cluster and the free ligand.

The MPI and ESP were calculated using the Multiwfn^[3]^ and Gaussian 16 software programs with the PBE0/ 6-31g method, SDD pseudopotential basis set is selected for Ti and Sn elements. According to the total energy, the most stable structure is selected, and the ESP is calculated to select the medium density standard. MPI analysis selects the default 0.001 a.u.electronic isosurface distribution characteristics.

# Supplementary Content


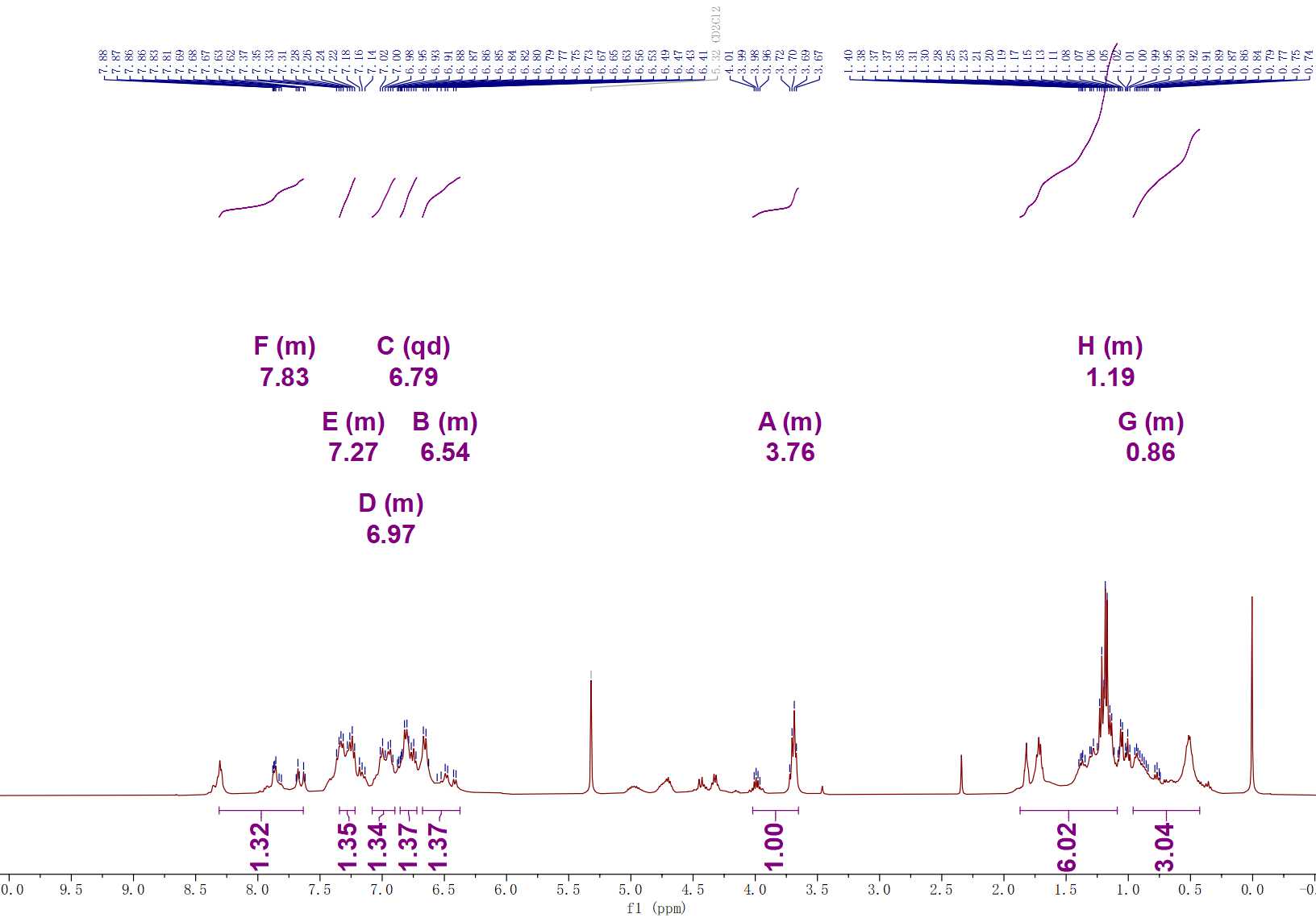


**Figure S1.** ^1^H NMR spectrum of the prepared **TS-1** in Methylene Chloride-*d*_2_.


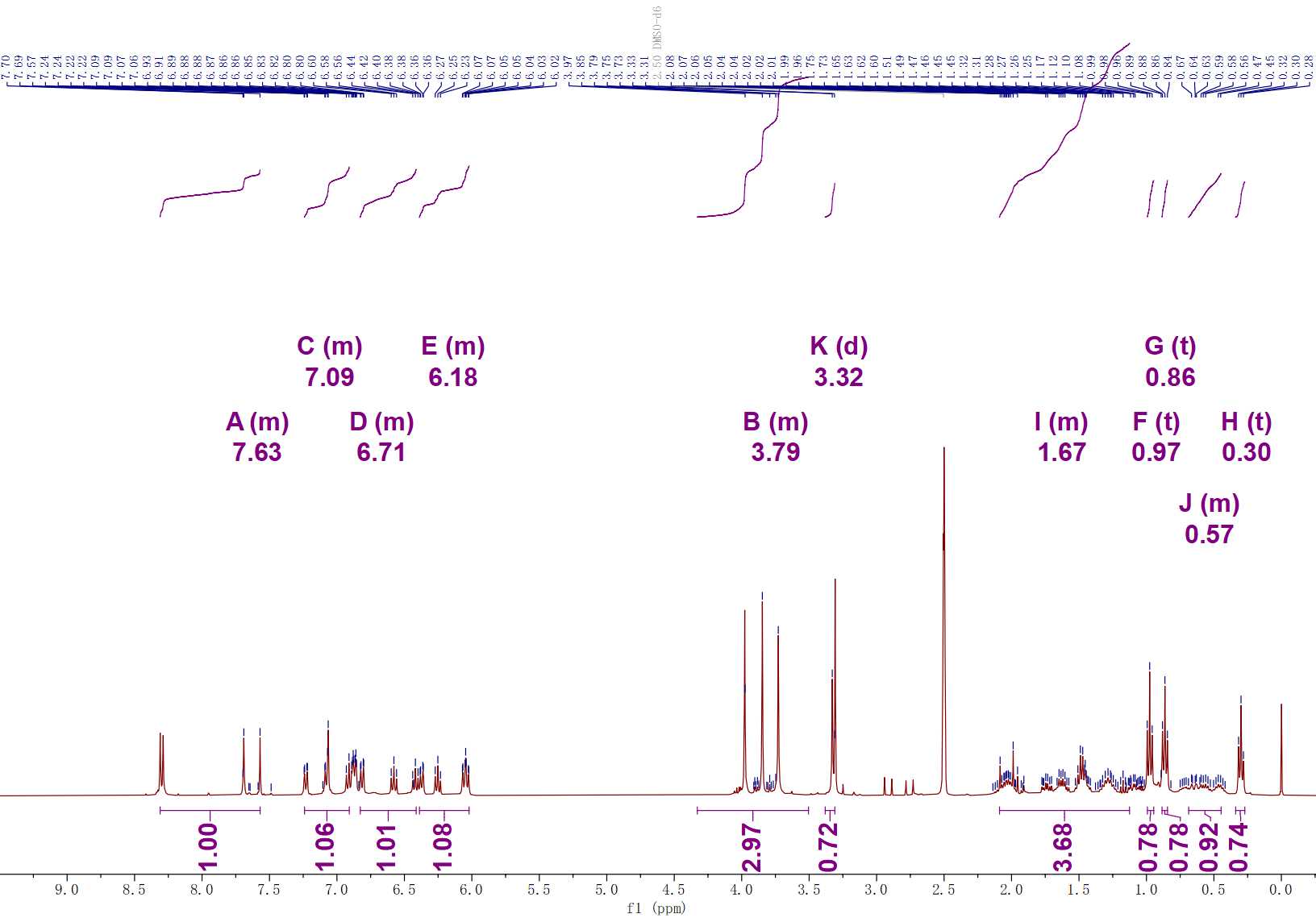


**Figure S2.** ^1^H NMR spectrum of the prepared **TS-2** in DMSO-*d*_6_.


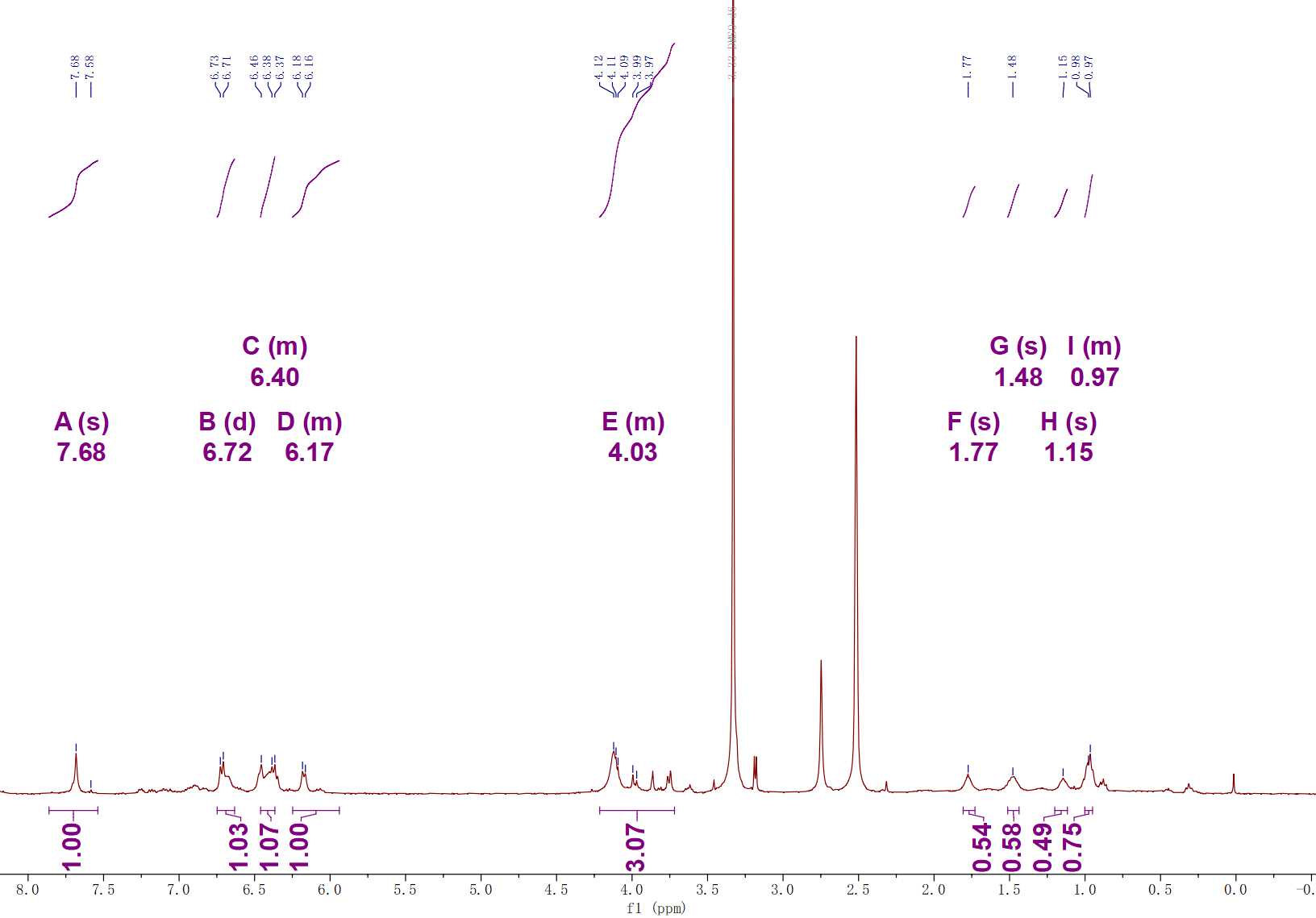


**Figure S3.** ^1^H NMR spectrum of the prepared **TS-3** in DMSO-*d*_6_.


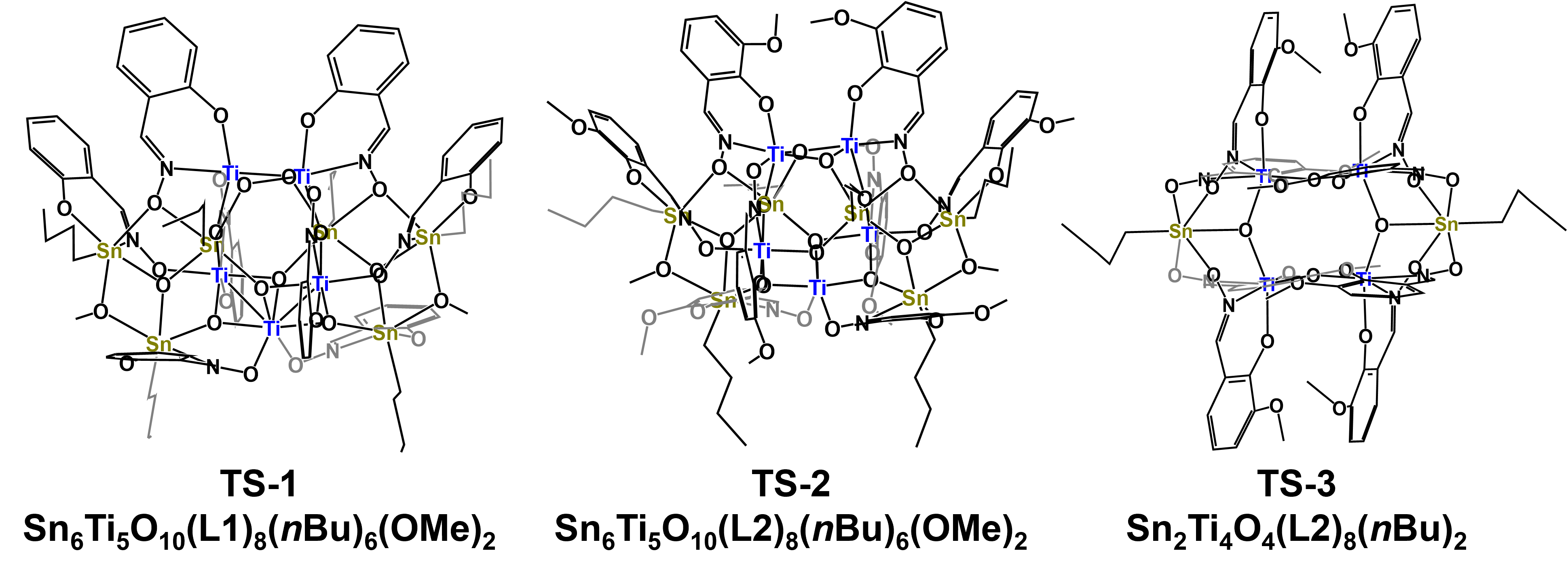


**Figure S4.** Chemical structure of the three metal oxo clusters, **TS-1** (Sn_6_Ti_5_O_10_(L1)_8_(*n*Bu)_6_(OMe)_2_), **TS-2** (Sn_6_Ti_5_O_10_(L2)_8_(*n*Bu)_6_(OMe)_2_), and **TS-3** (Sn_2_Ti_4_O_4_(L2)_8_(*n*Bu)_2_).


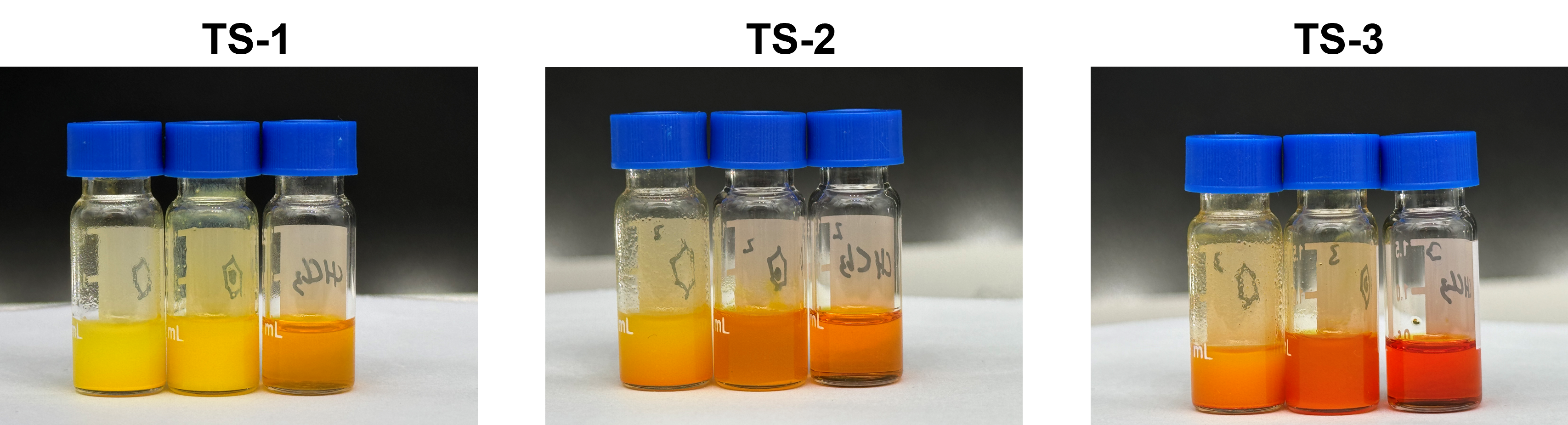


**Figure S5.** Solubility of **TS-1**, **TS-2**, and **TS-3** in different solvents (from left to right: cyclohexane, benzene, chloroform).


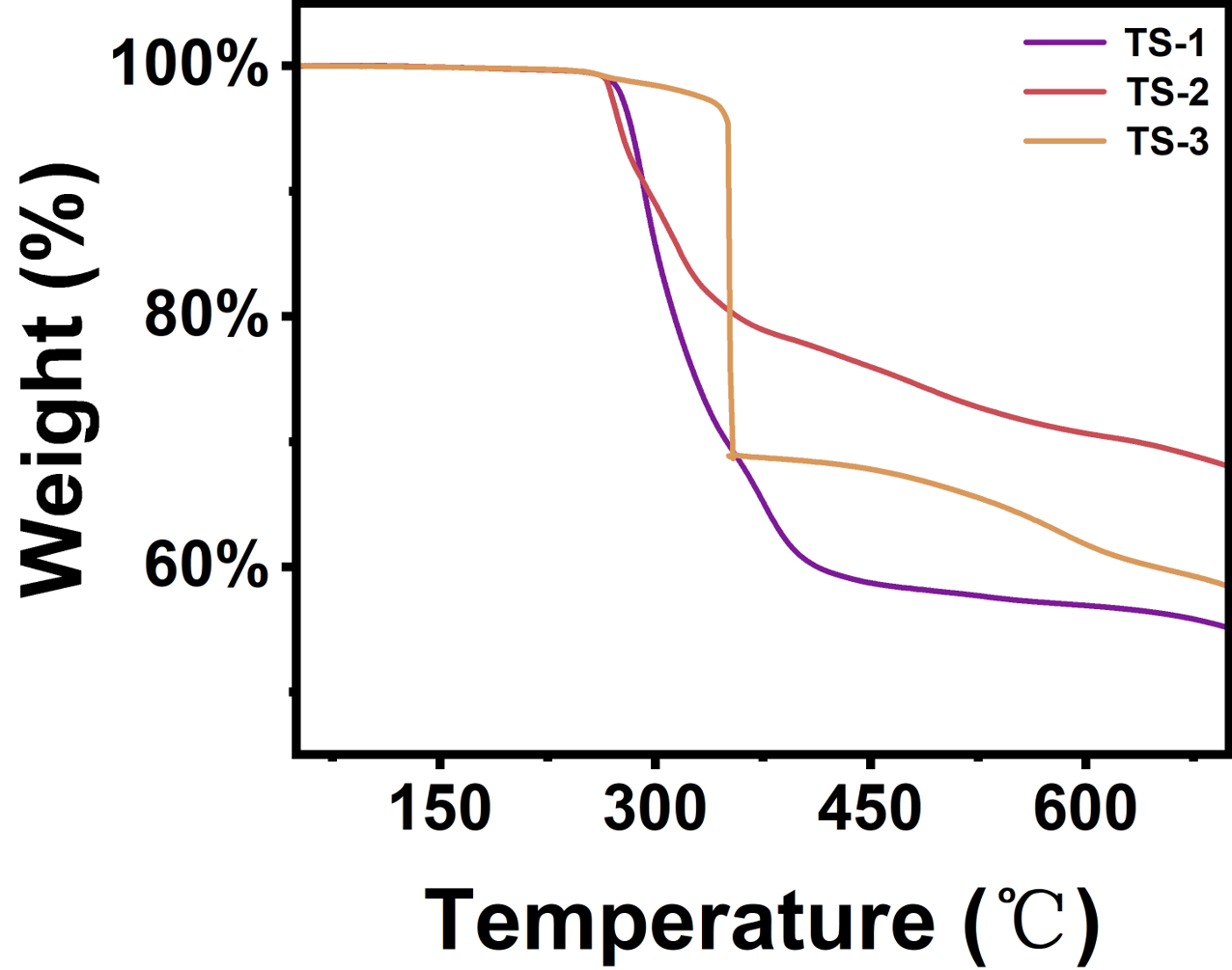


**Figure S6.** TGA curve of the prepared **TS-1**, **TS-2**, and **TS-3** under Ar atmosphere.


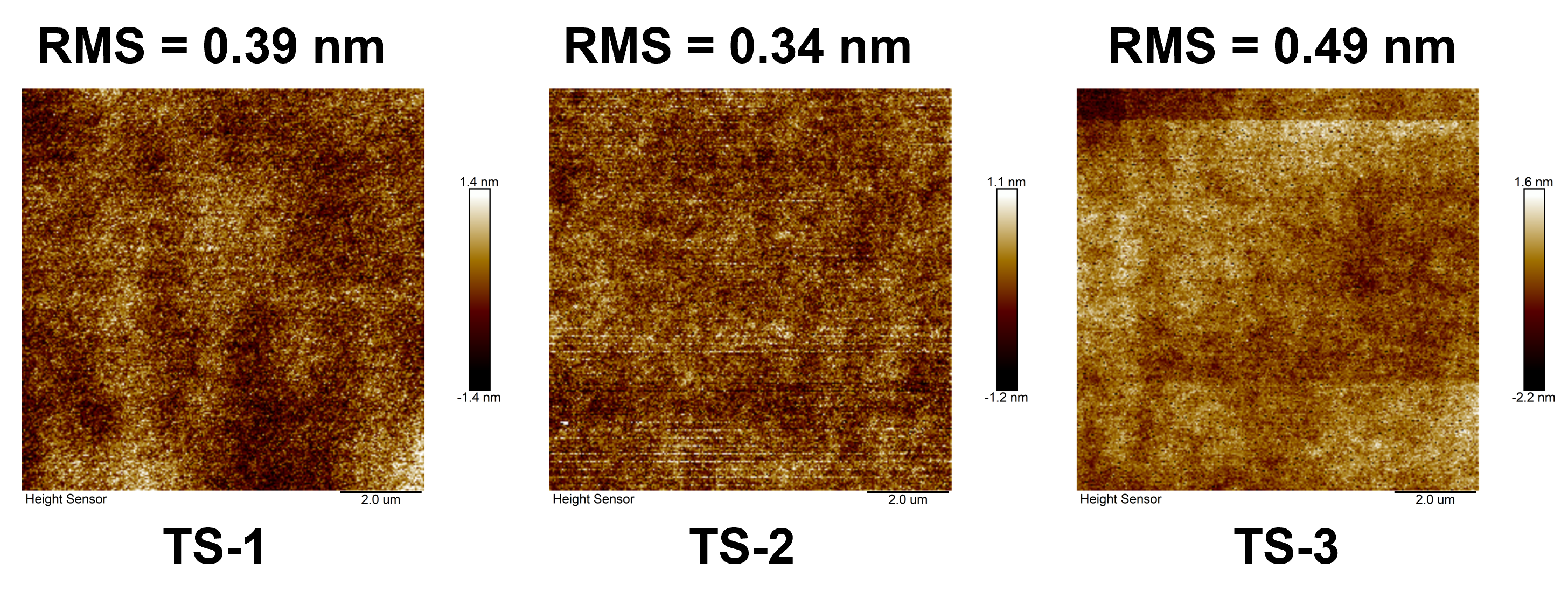


**Figure S7.** Root Mean Square roughness (RMS) of **TS-1**, **TS-2**, and **TS-3** at 8 mg/mL and 4000 r/min, measured by AFM.


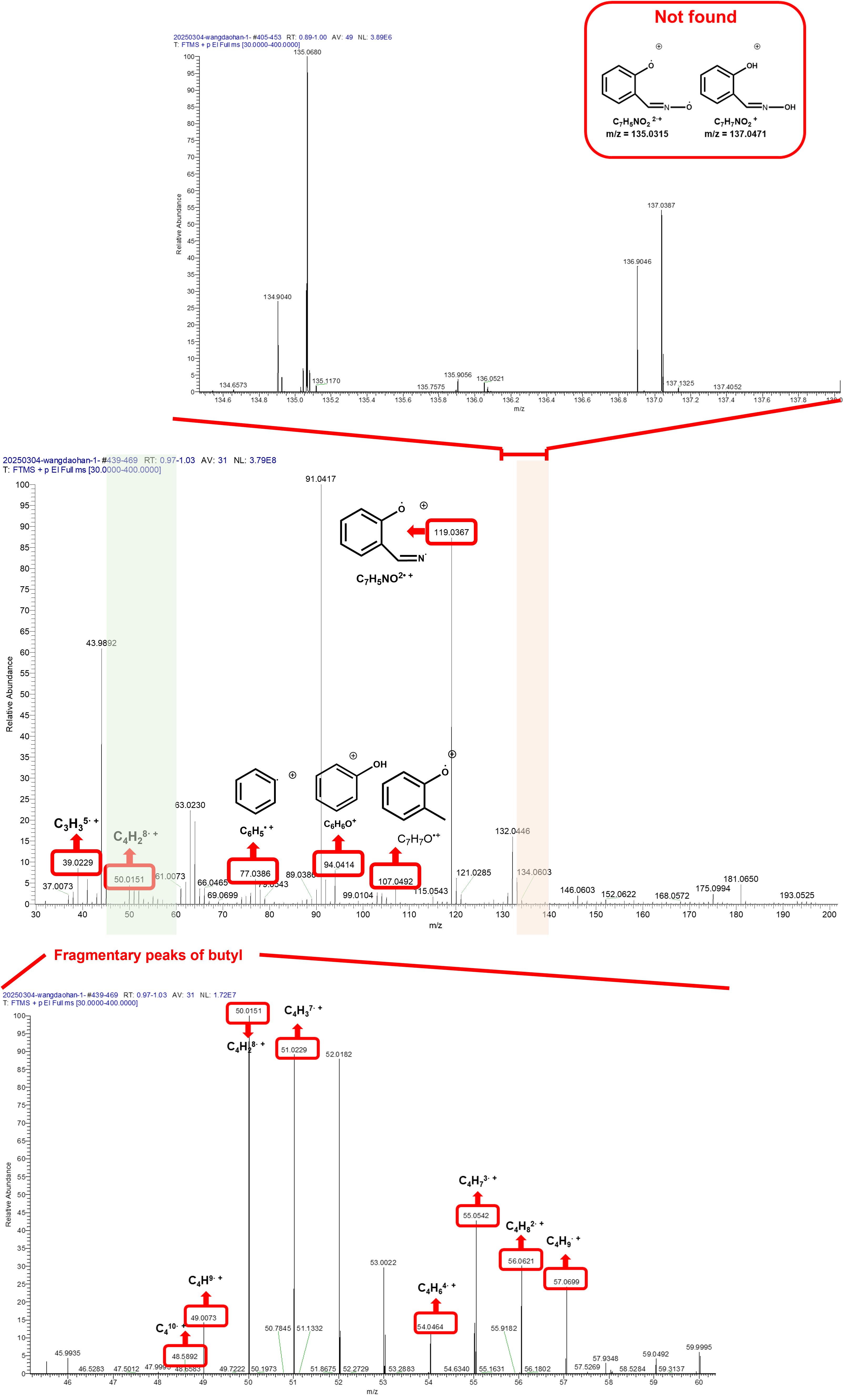


**Figure S8.** DIP-MS of **TS-1** obtained under EI mode.


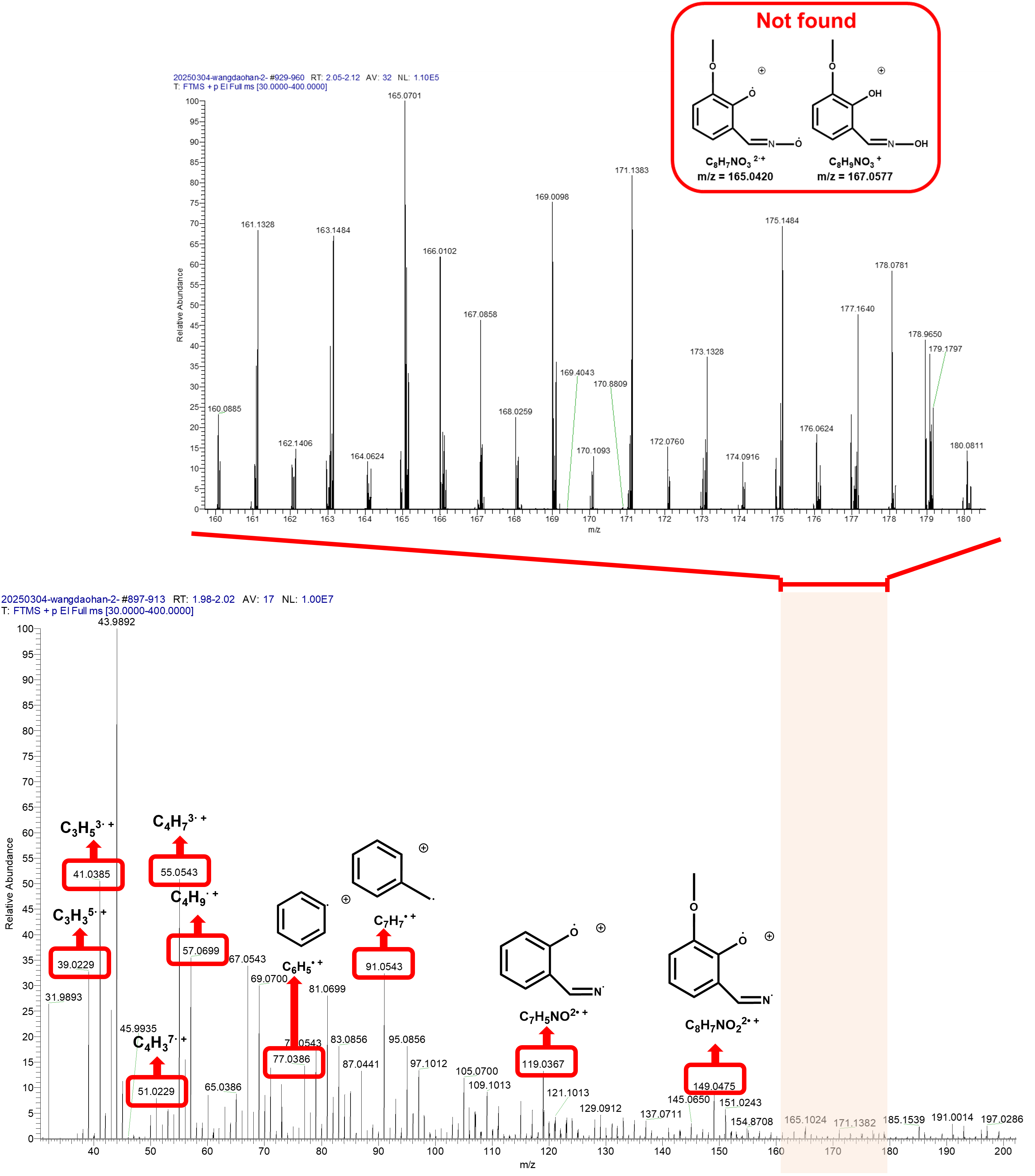


**Figure S9.** DIP-MS of **TS-2** obtained under EI mode.


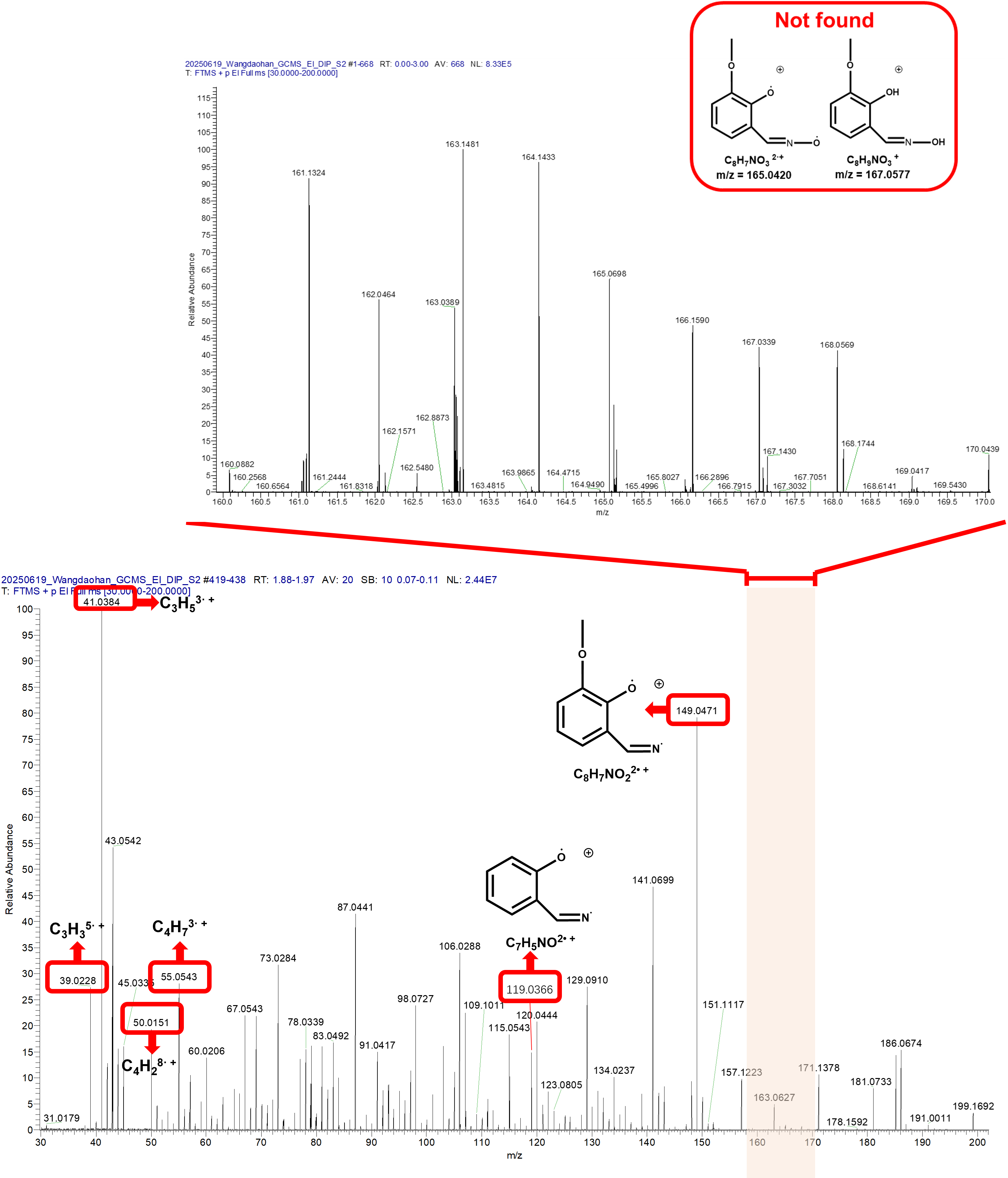


**Figure S10.** DIP-MS of **TS-3** obtained under EI mode.


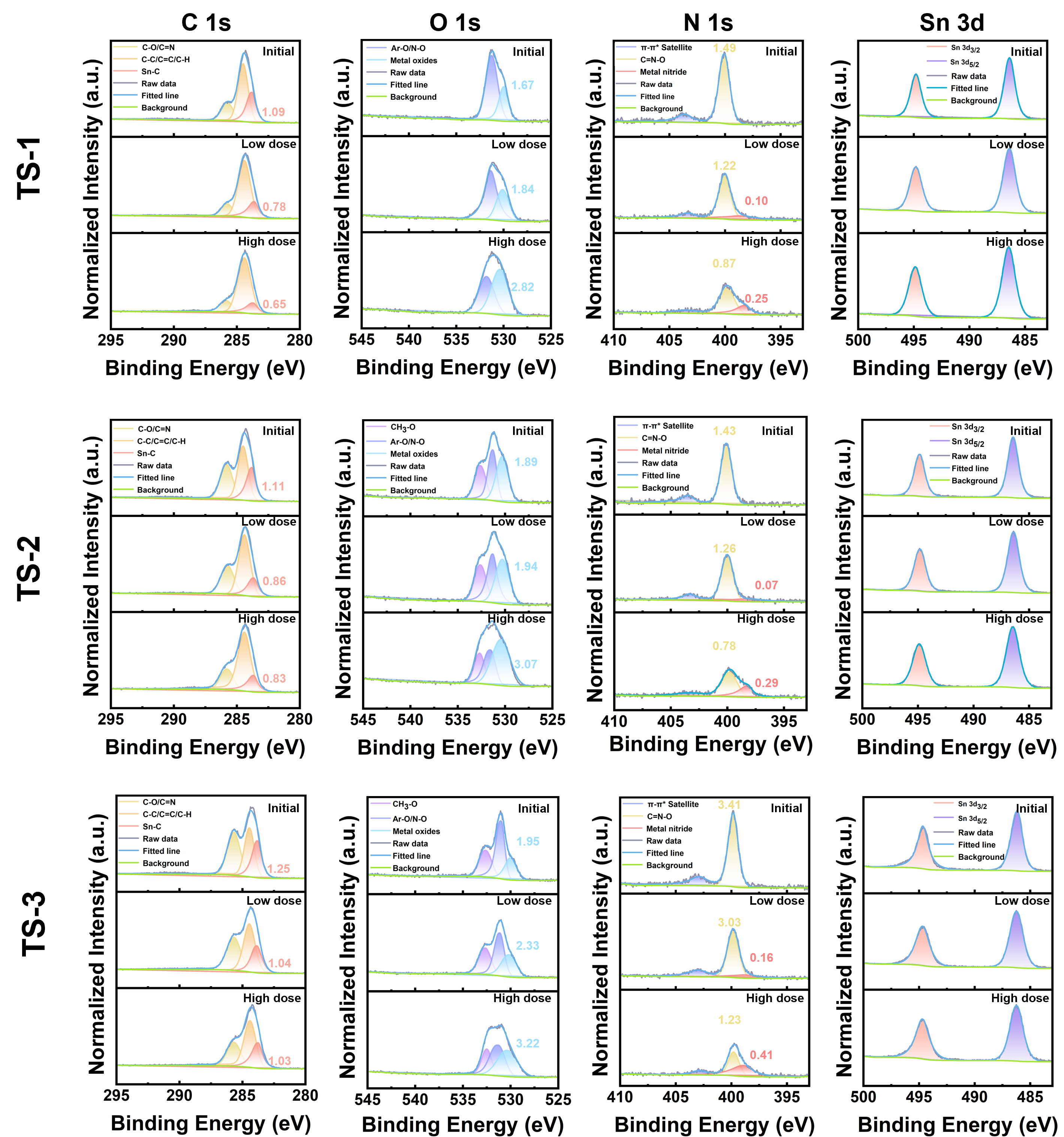


**Figure S11.** High-resolution XPS spectra of C 1s, O 1s, N 1s, and Sn 3d of **TS-1**, **TS-2**, and **TS-3**, under different dose of EBL.


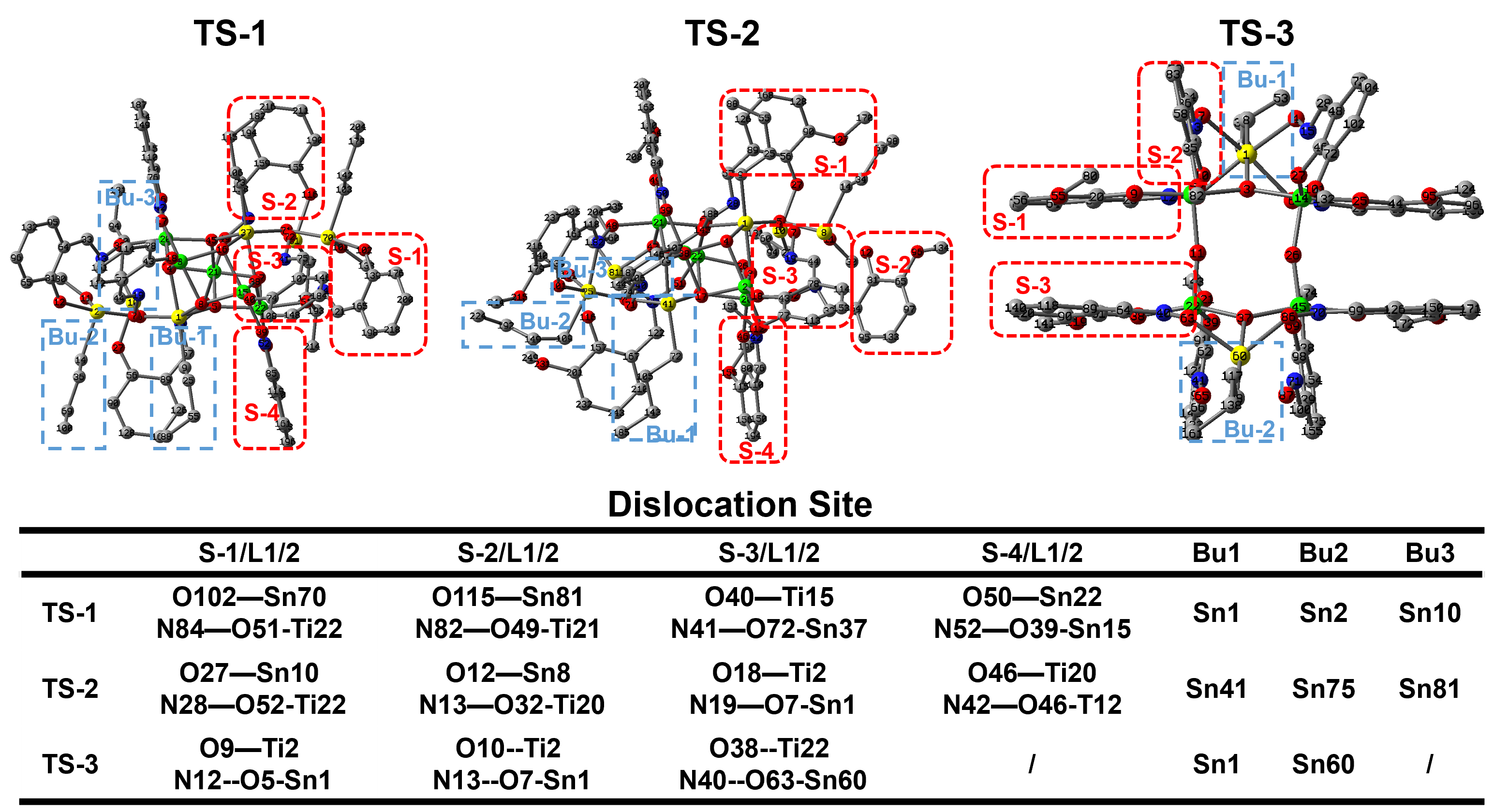


**Figure S12.** Schematic Diagram of Ligand Dissociation Positions in **TS-1**, **TS-2**, and **TS-3** Clusters.


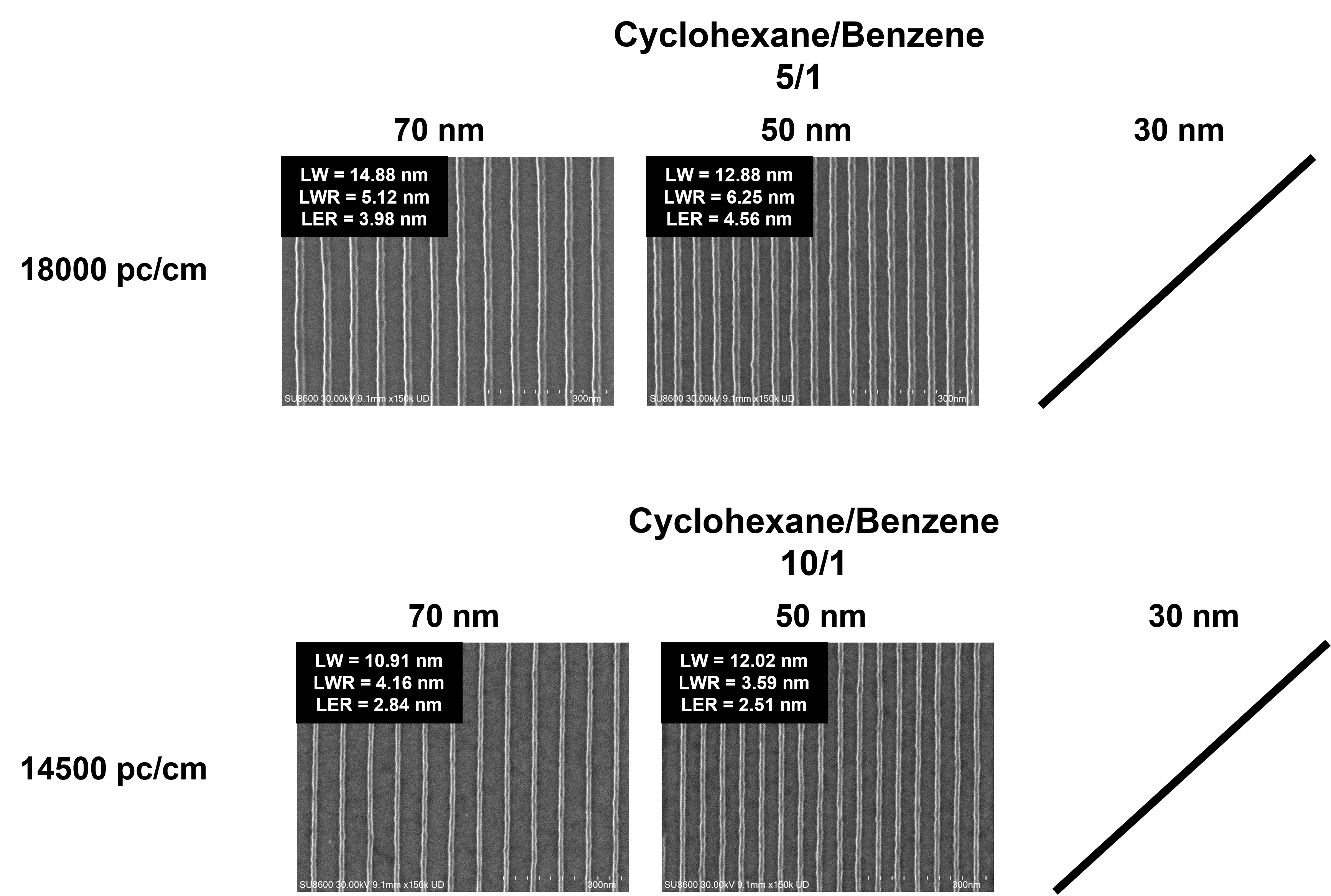


**Figure S13.** Patterns obtained by developing the exposed **TS-1** with different volume ratios of cyclohexane and benzene.


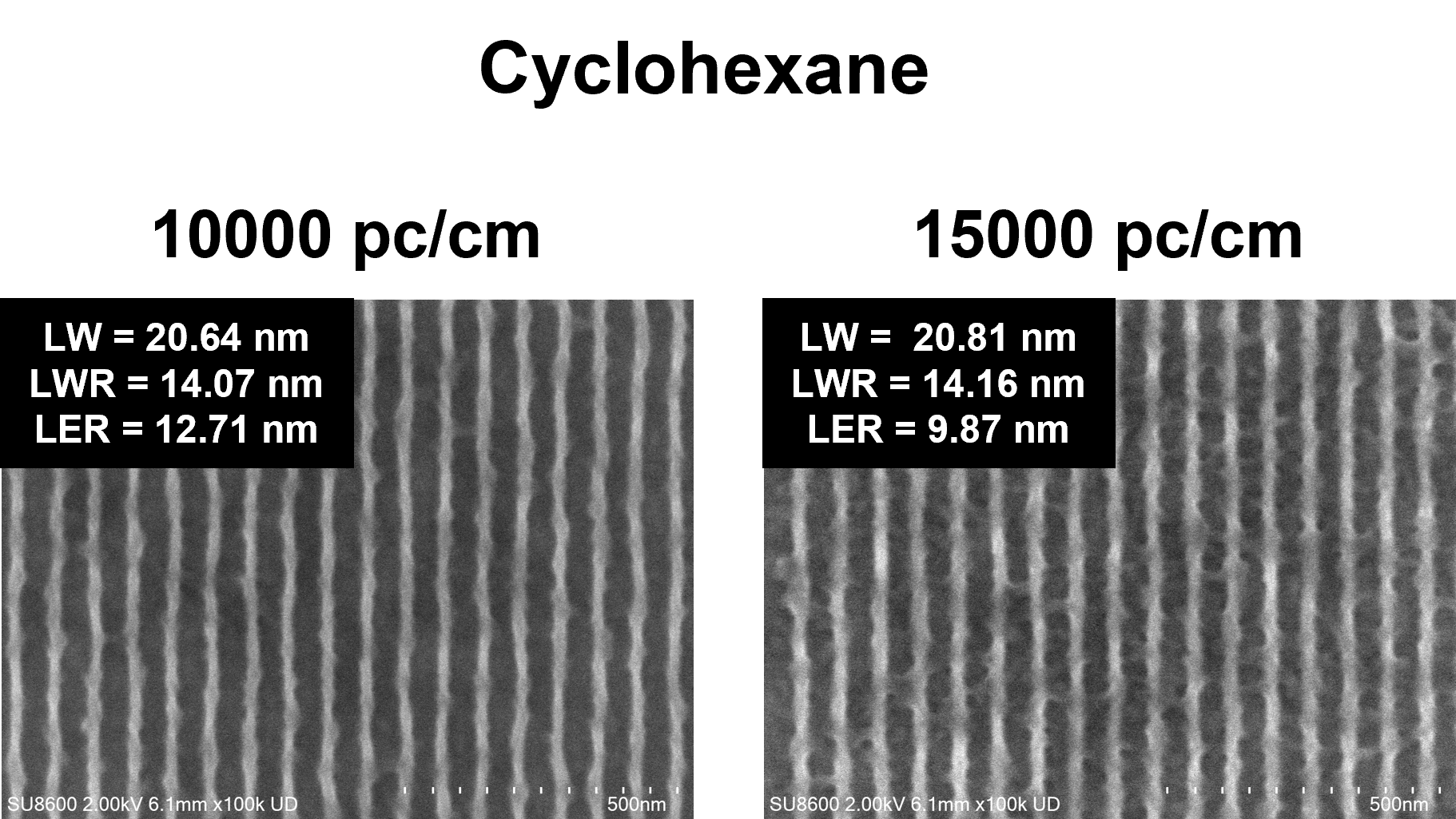


**Figure S14.** The pitch of 70 nm lines obtained by developing the exposed **TS-1** with cyclohexane.


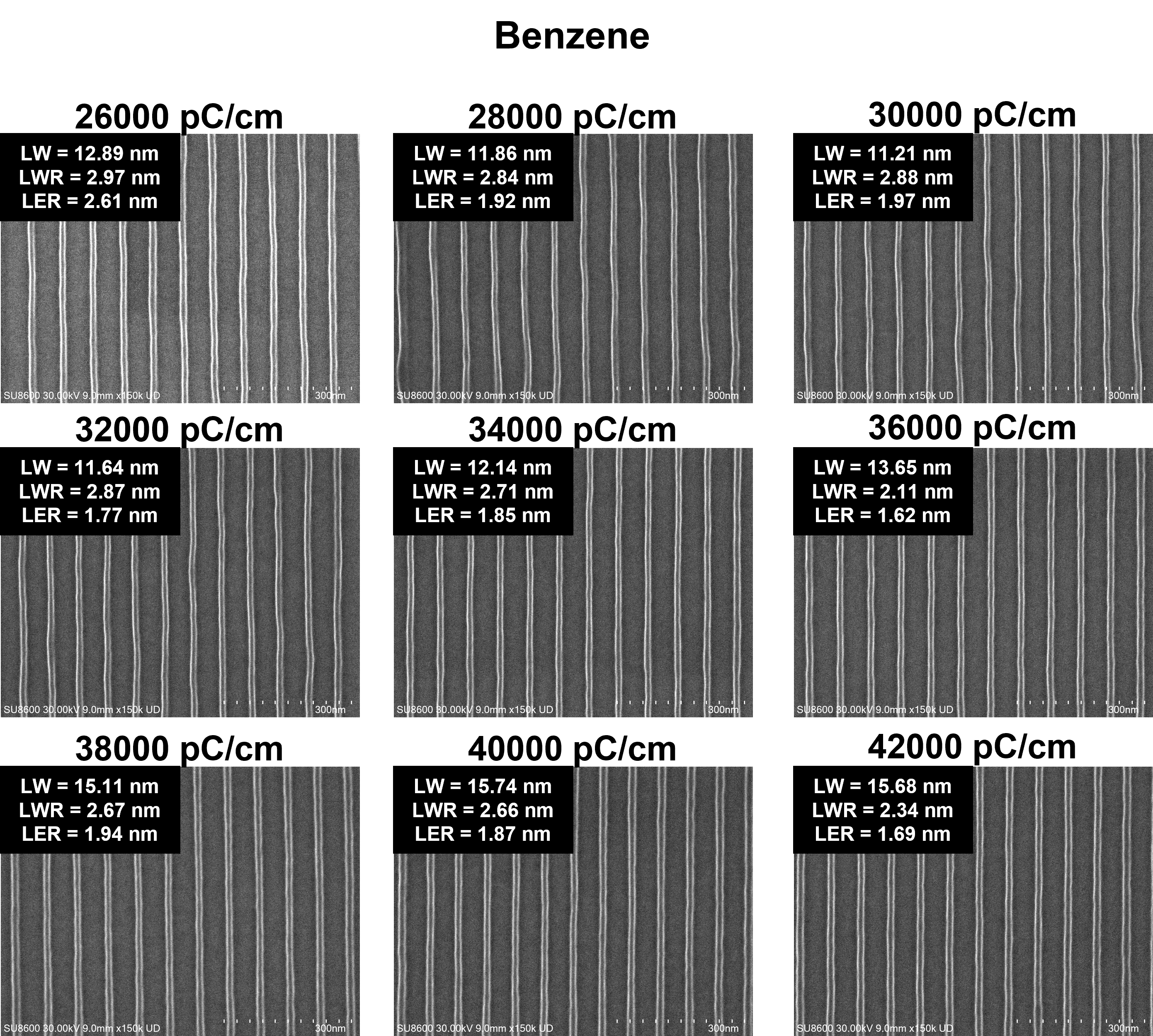


**Figure S15.** The pitch of 70 nm lines obtained by developing the exposed **TS-1** with benzene.


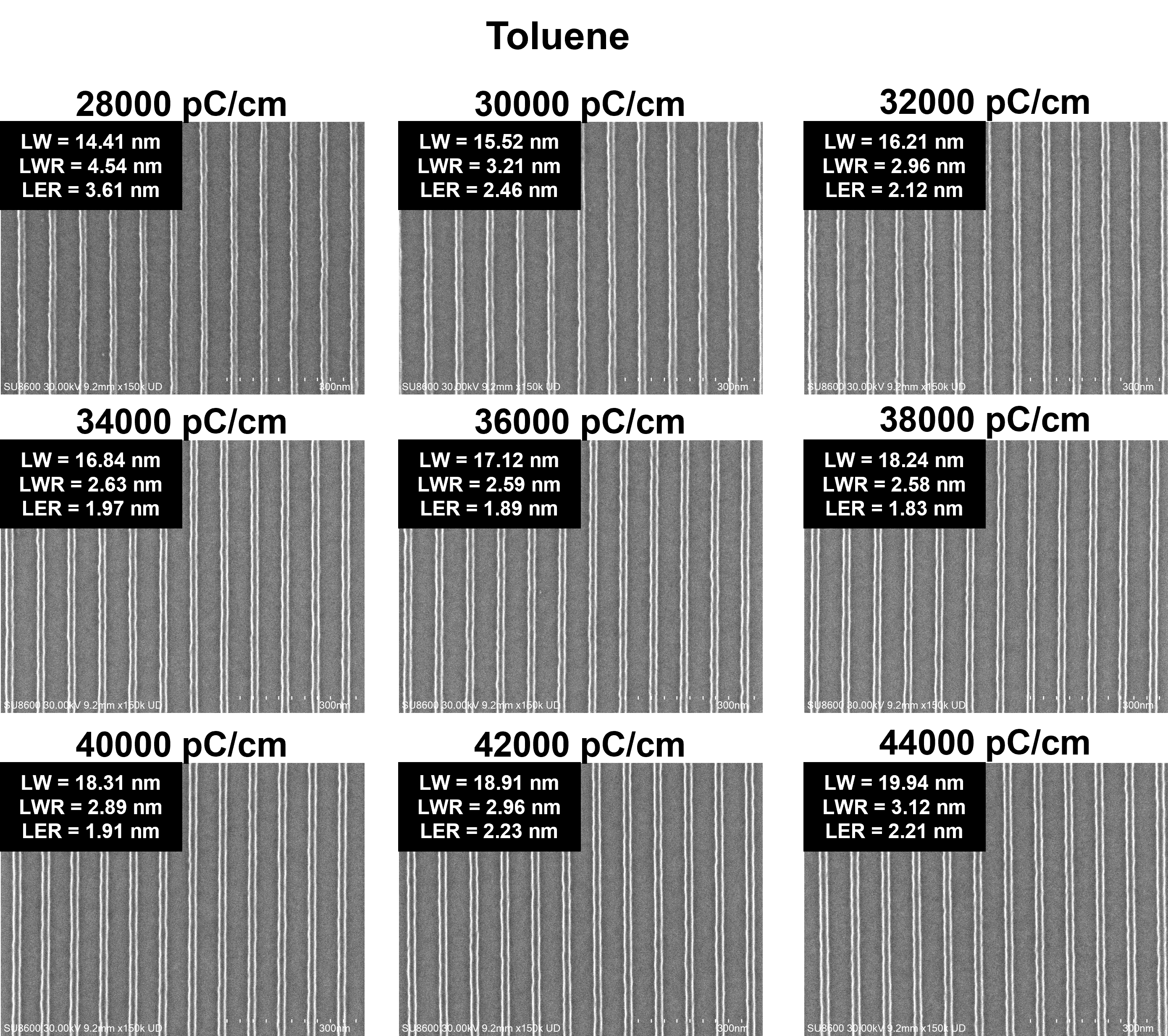


**Figure S16.** The pitch of 70 nm lines obtained by developing the exposed **TS-1** with toluene.


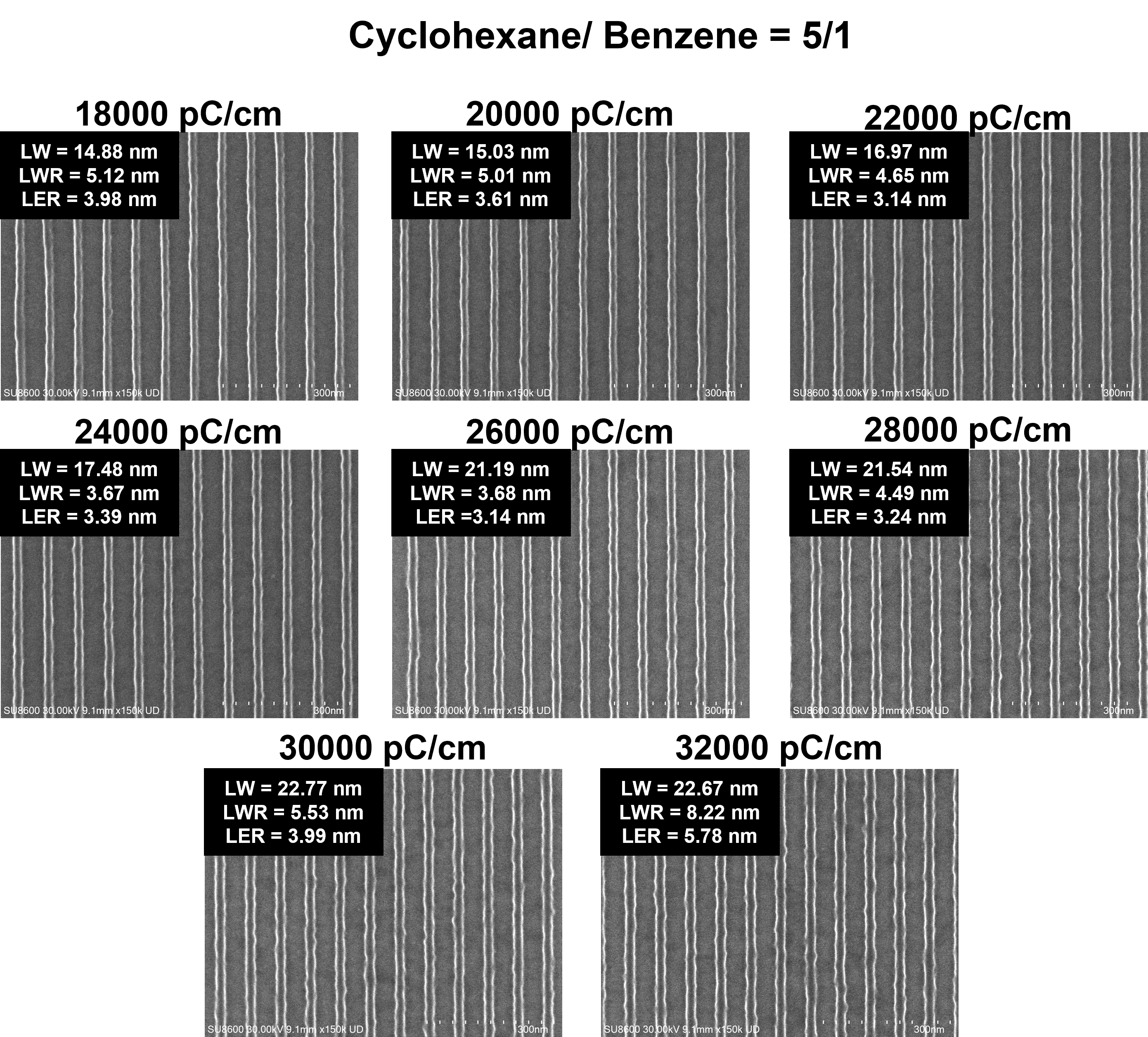


**Figure S17.** The pitch of 70 nm lines obtained by developing the exposed **TS-1** with cyclohexane/benzene = 5/1.


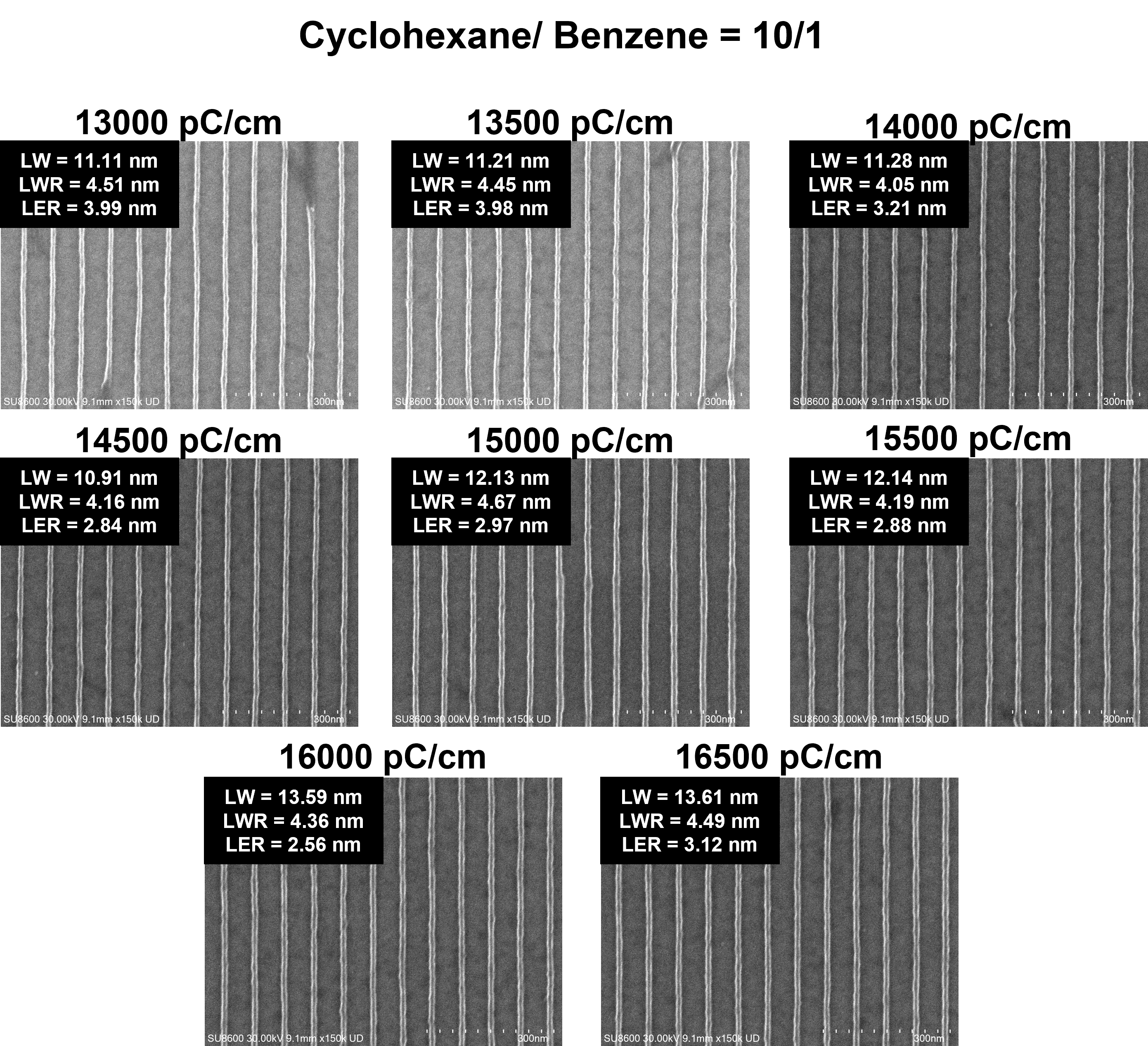


**Figure S18.** The pitch of 70 nm lines obtained by developing the exposed **TS-1** with cyclohexane/benzene = 10/1.


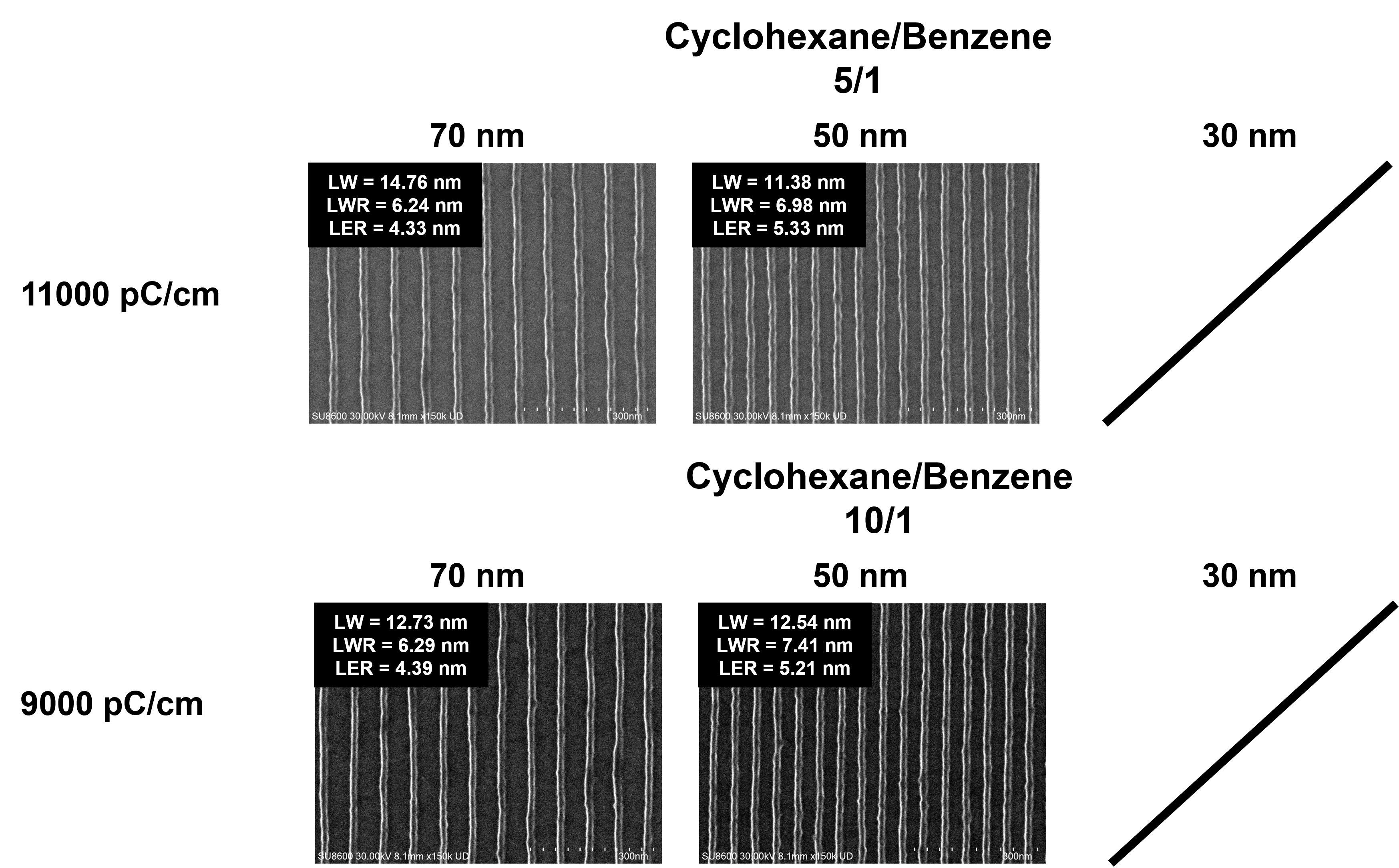


**Figure S19.** Patterns obtained by developing the exposed **TS-2** with different volume ratios of cyclohexane and benzene.


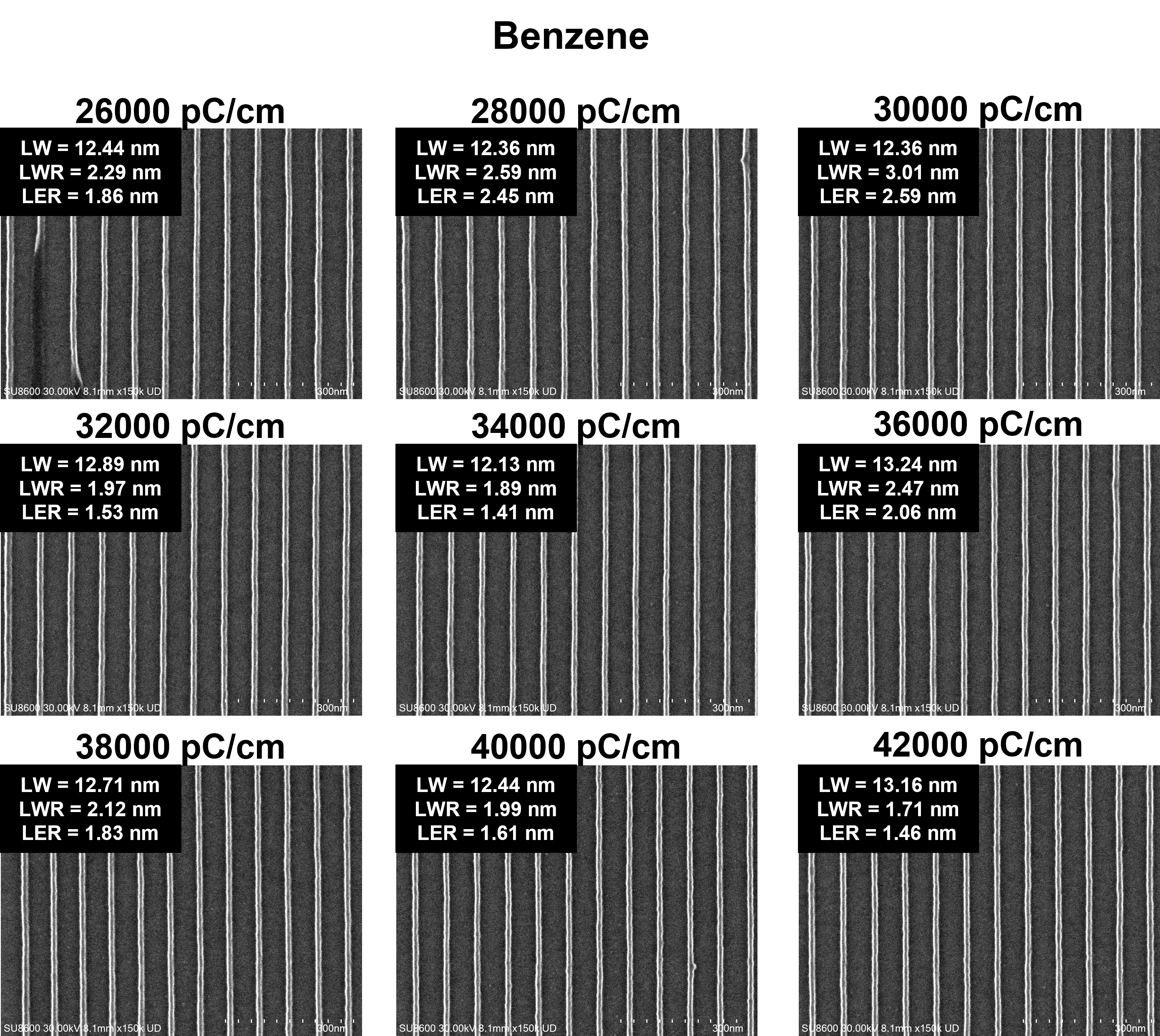


**Figure S20.** The pitch of 70 nm lines obtained by developing the exposed **TS-2** with benzene.


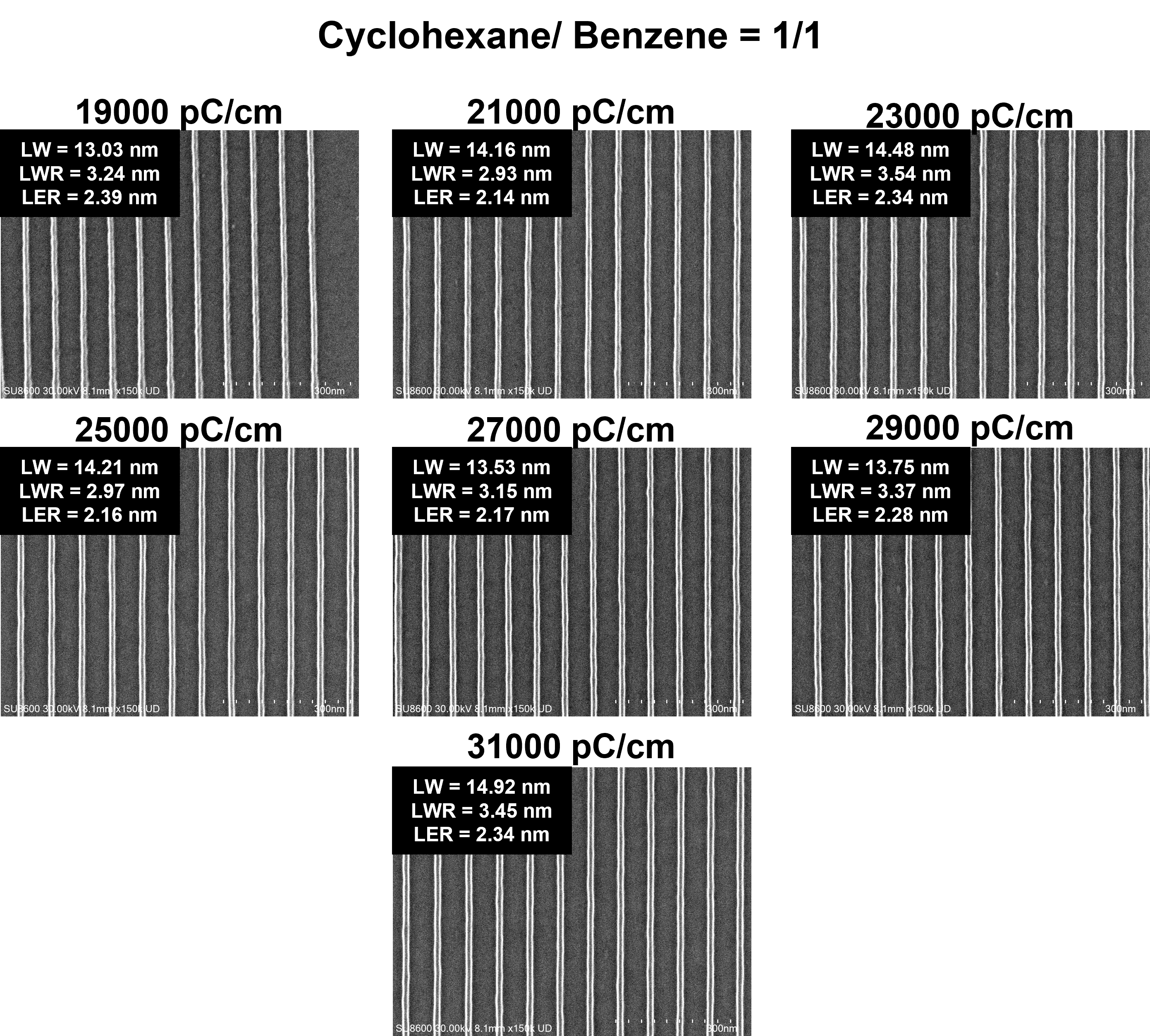


**Figure S21.** The pitch of 70 nm lines obtained by developing the exposed **TS-2** with cyclohexane/benzene = 1/1.


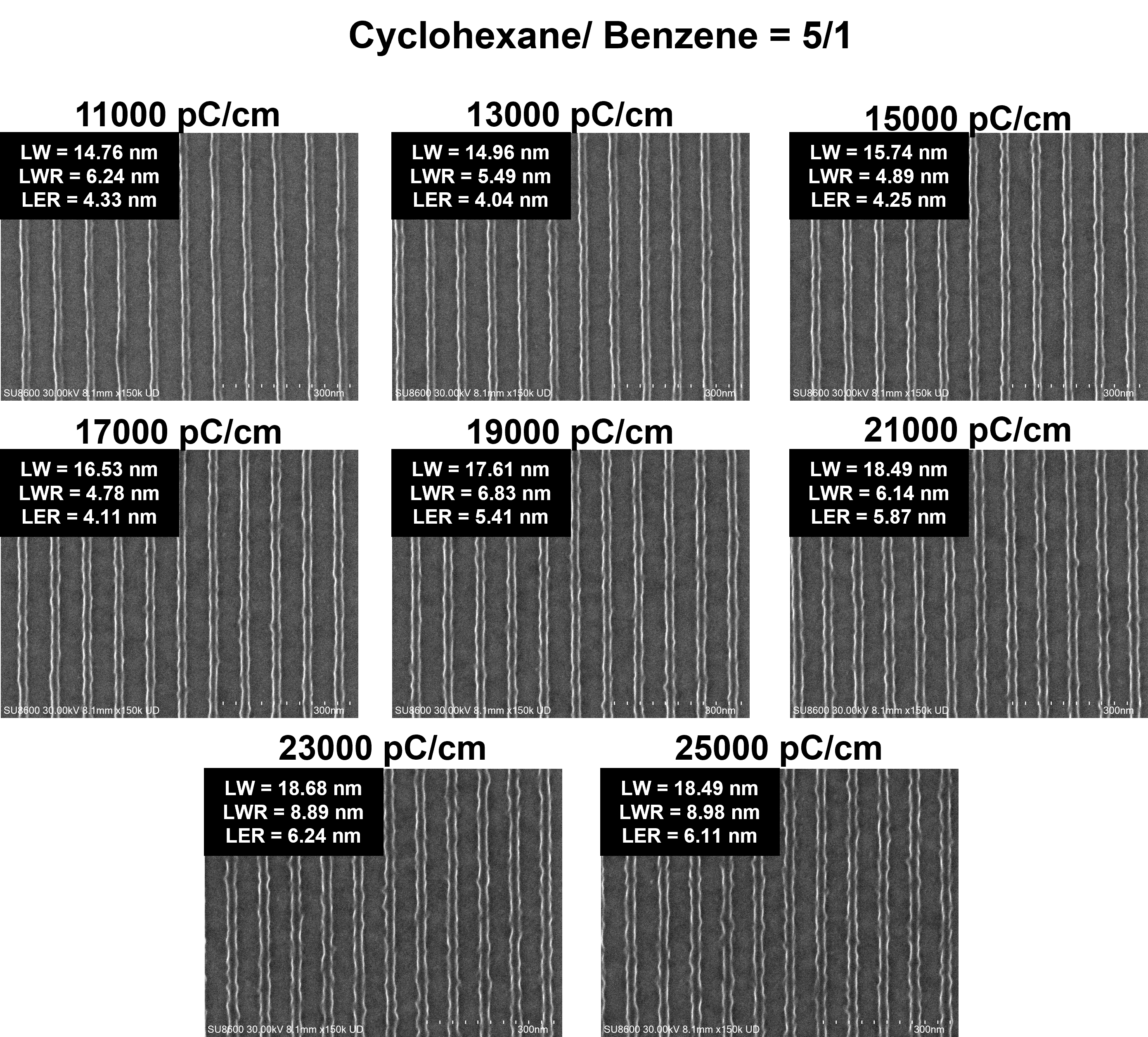


**Figure S22.** The pitch of 70 nm lines obtained by developing the exposed **TS-2** with cyclohexane/benzene = 5/1.


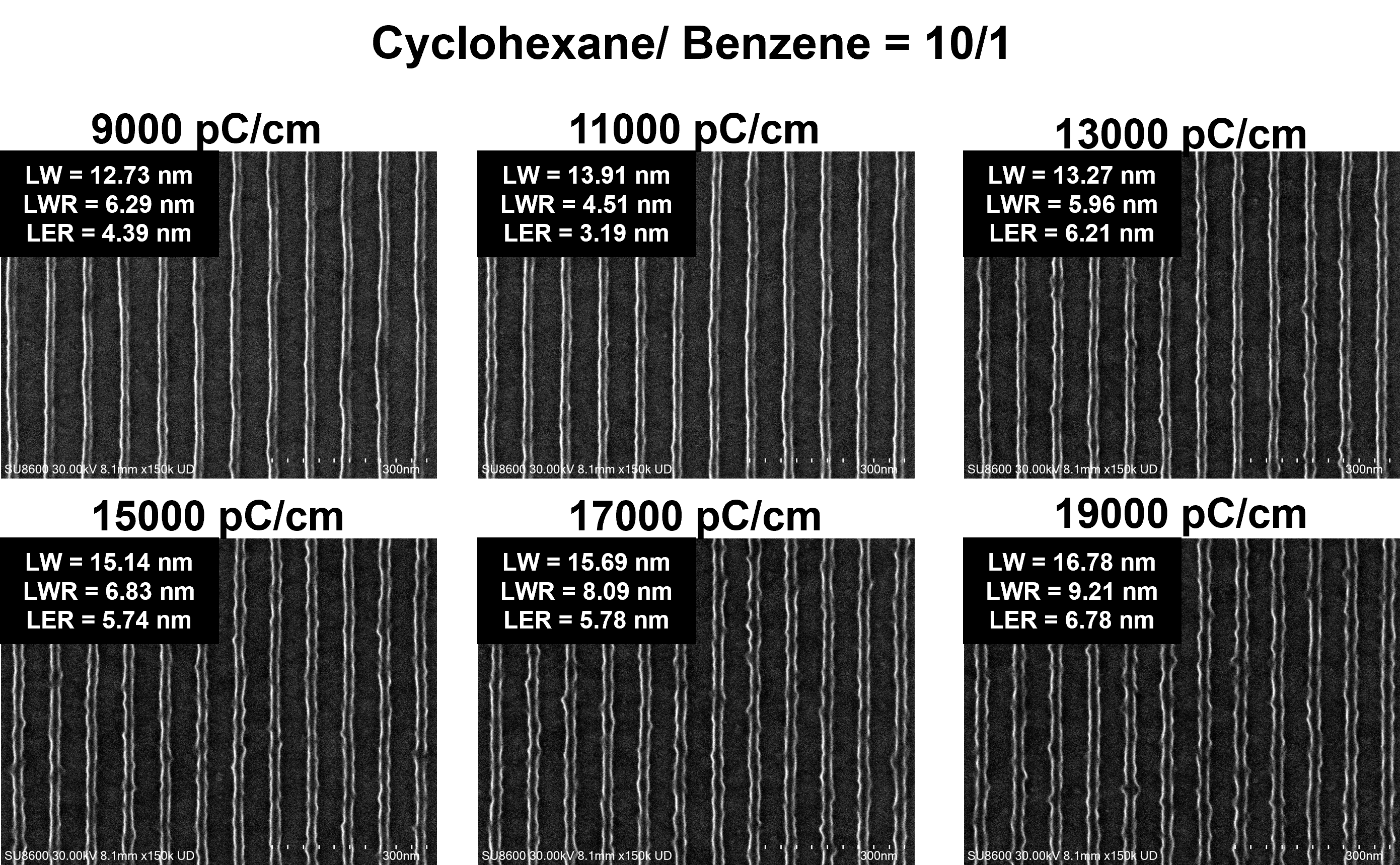


**Figure S23.** The pitch of 70 nm lines obtained by developing the exposed **TS-2** with cyclohexane/benzene = 10/1.


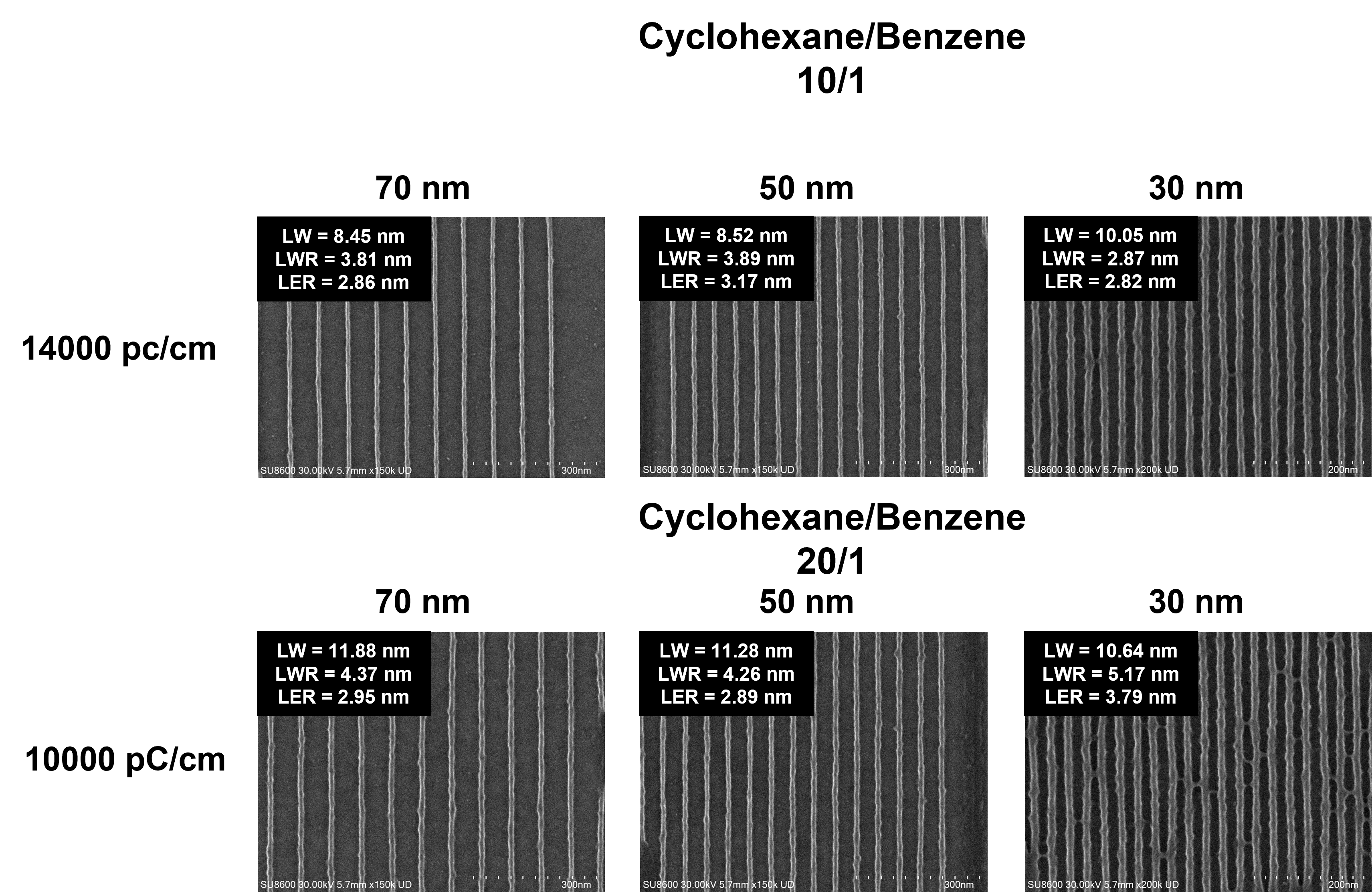


**Figure S24.** Patterns obtained by developing the exposed **TS-3** with different volume ratios of cyclohexane and benzene.


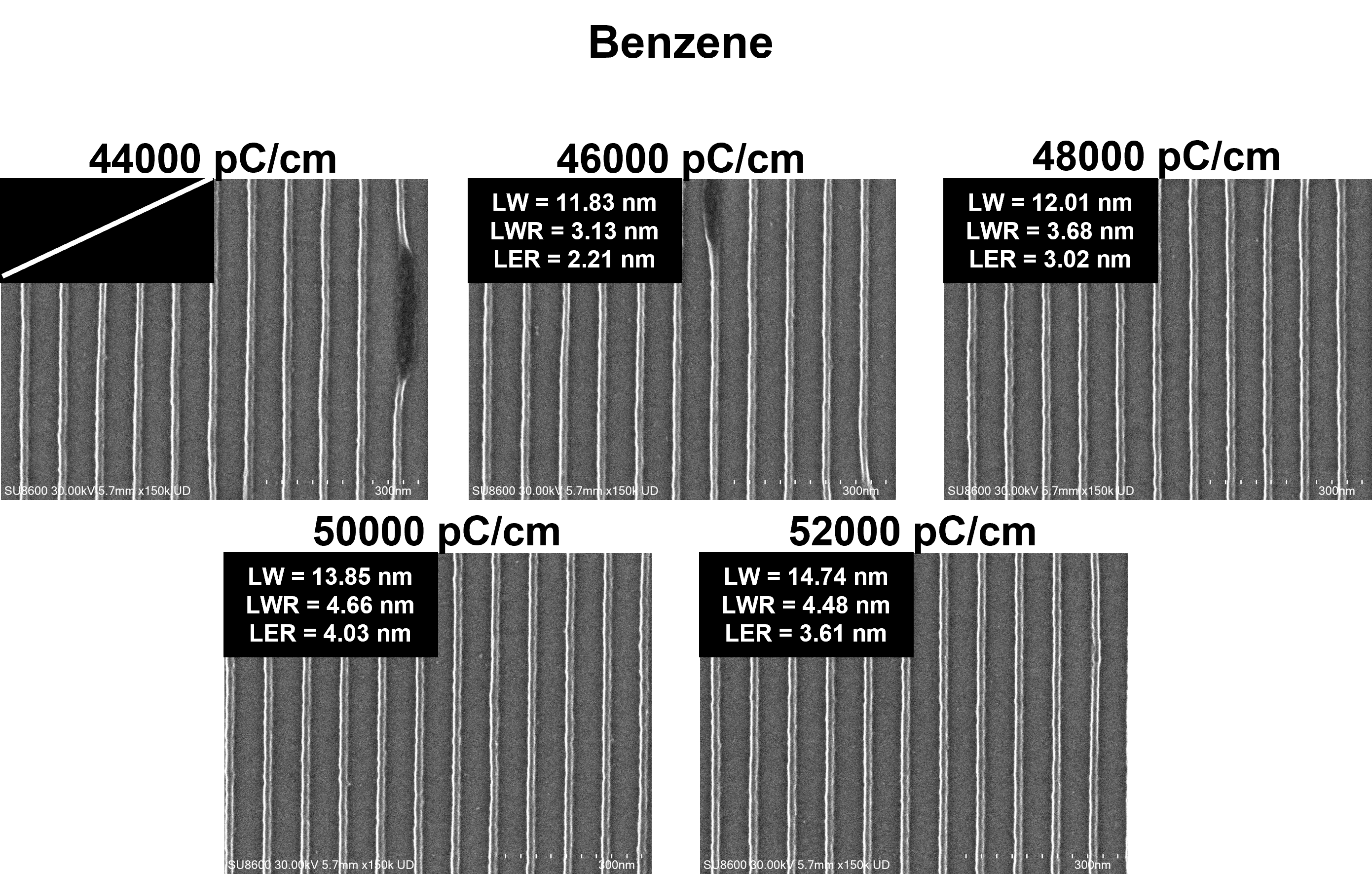


**Figure S25.** The pitch of 70 nm lines obtained by developing the exposed **TS-3** with benzene.


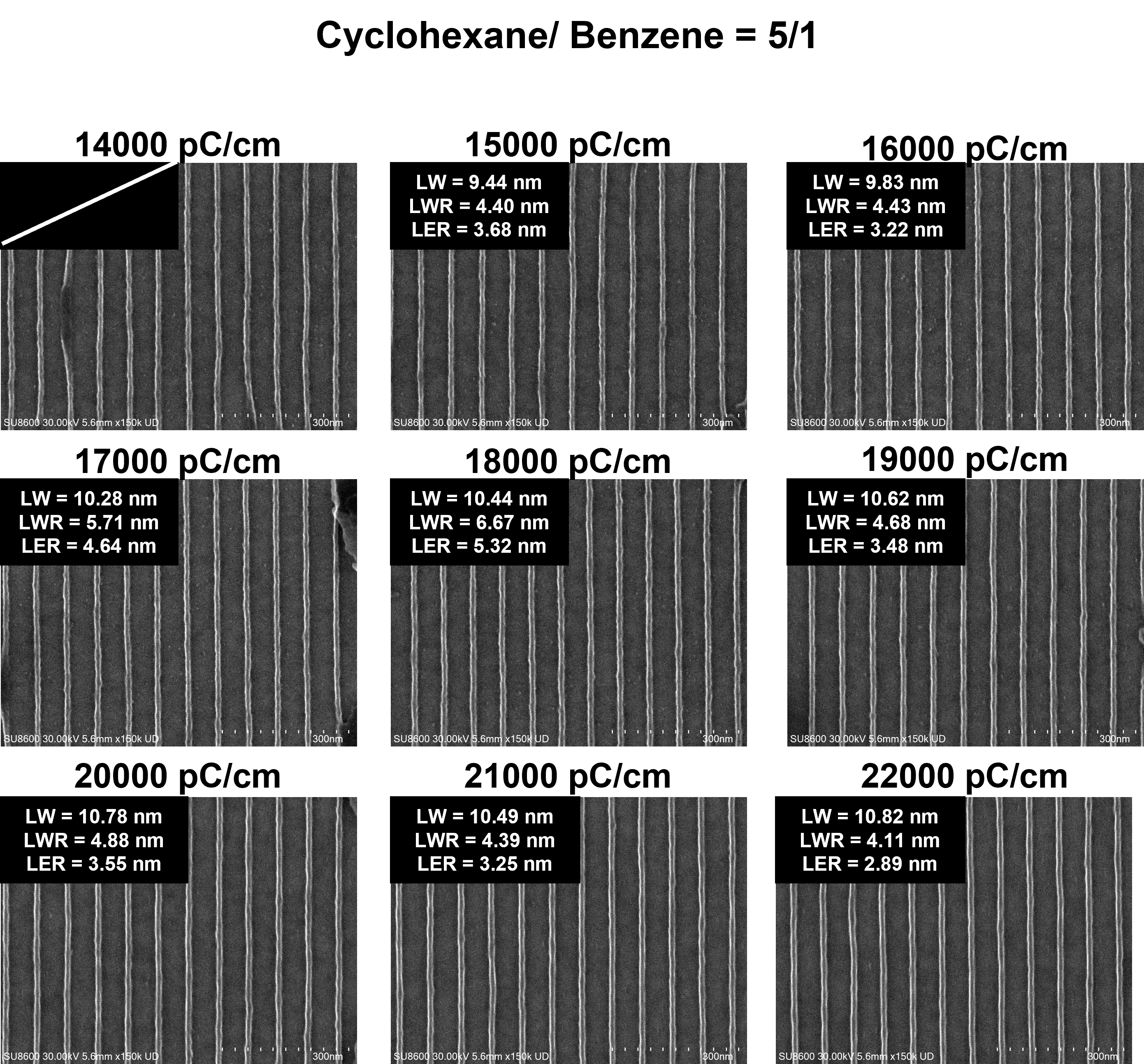


**Figure S26.** The pitch of 70 nm lines obtained by developing the exposed **TS-3** with cyclohexane/benzene = 5/1.


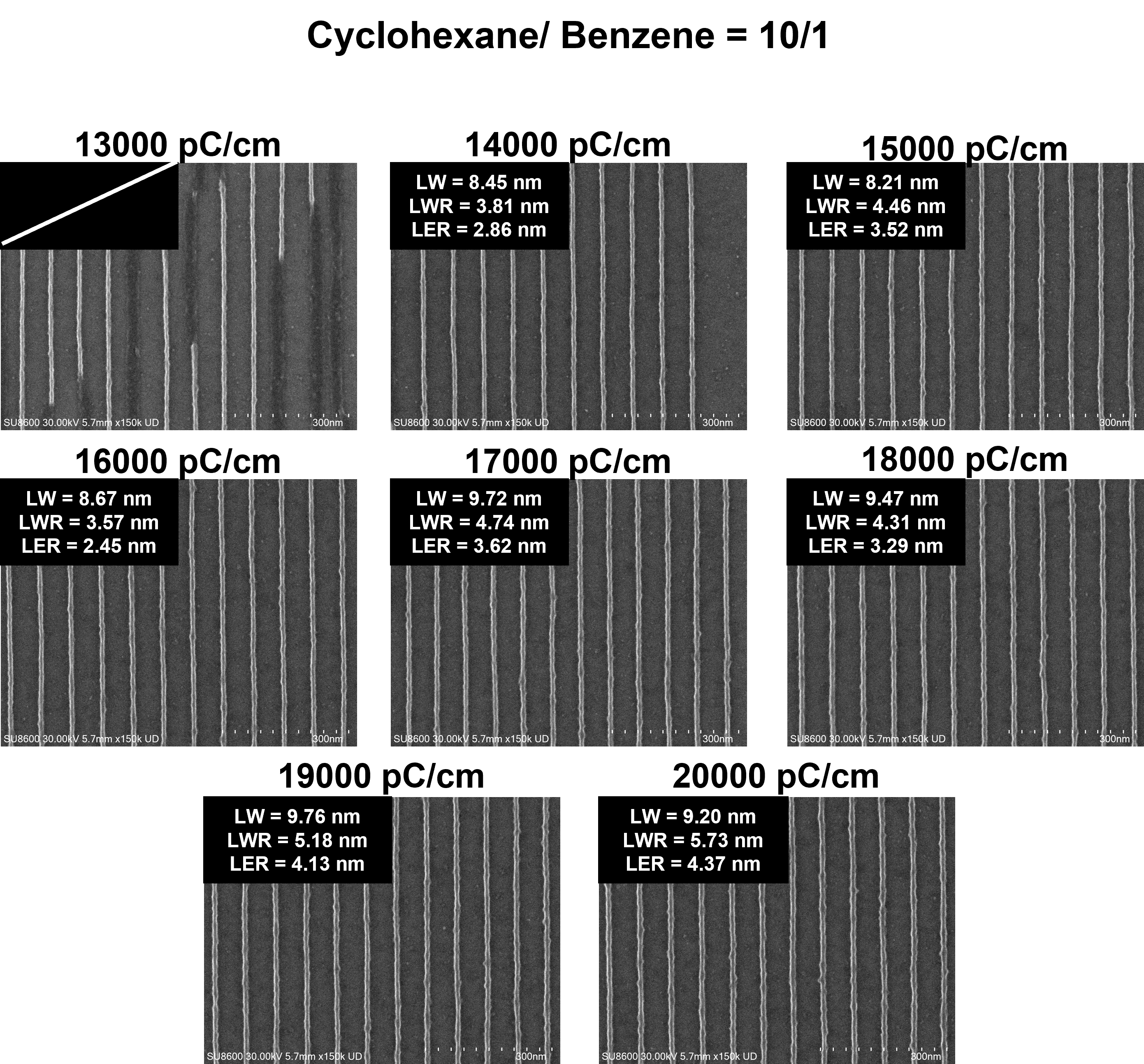


**Figure S27.** The pitch of 70 nm lines obtained by developing the exposed **TS-3** with cyclohexane/benzene = 10/1.


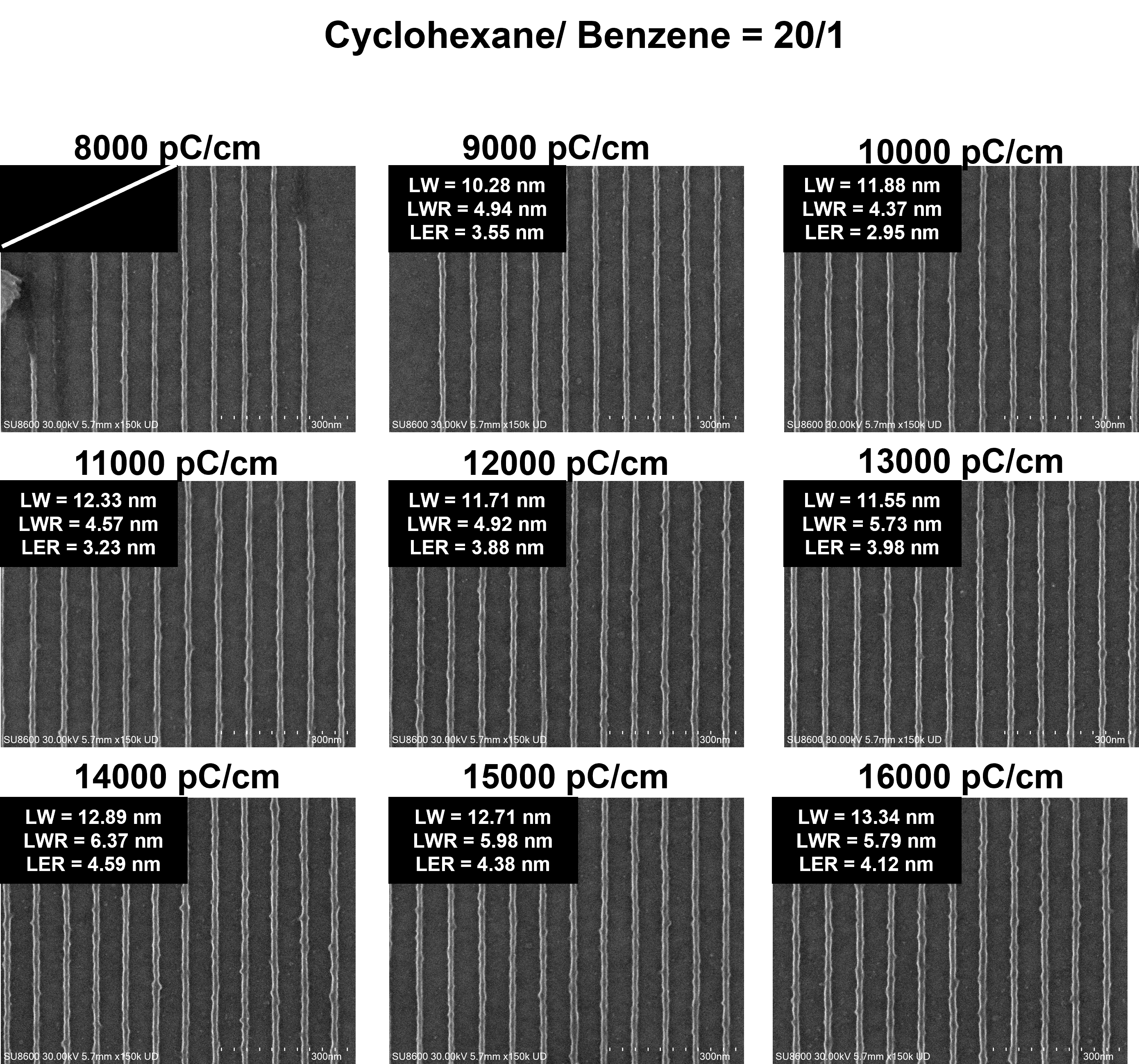


**Figure S28.** The pitch of 70 nm lines obtained by developing the exposed **TS-3** with cyclohexane/benzene = 20/1.


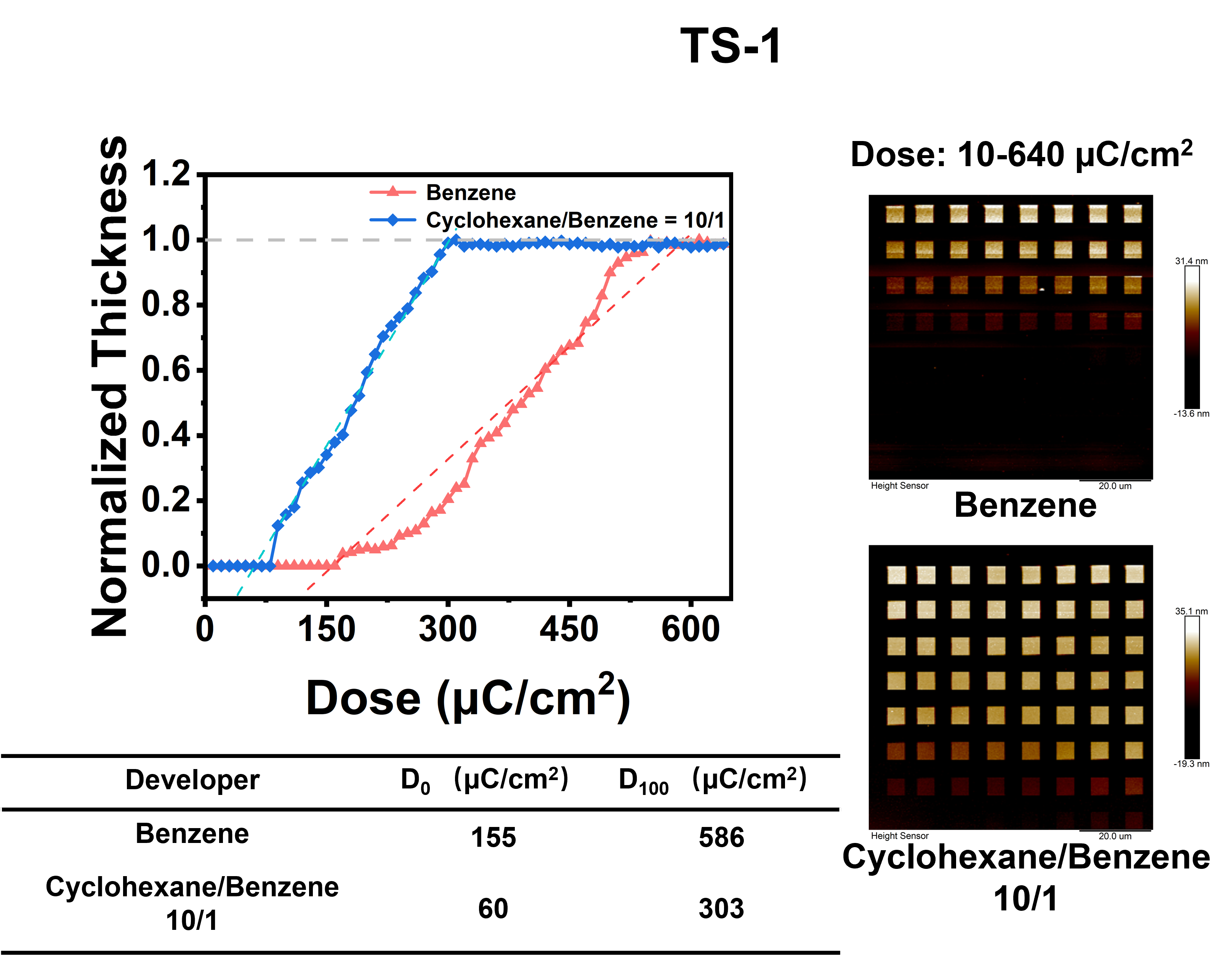


**Figure S29.** Dose array and contrast curve of **TS-1** before and after developer optimization.


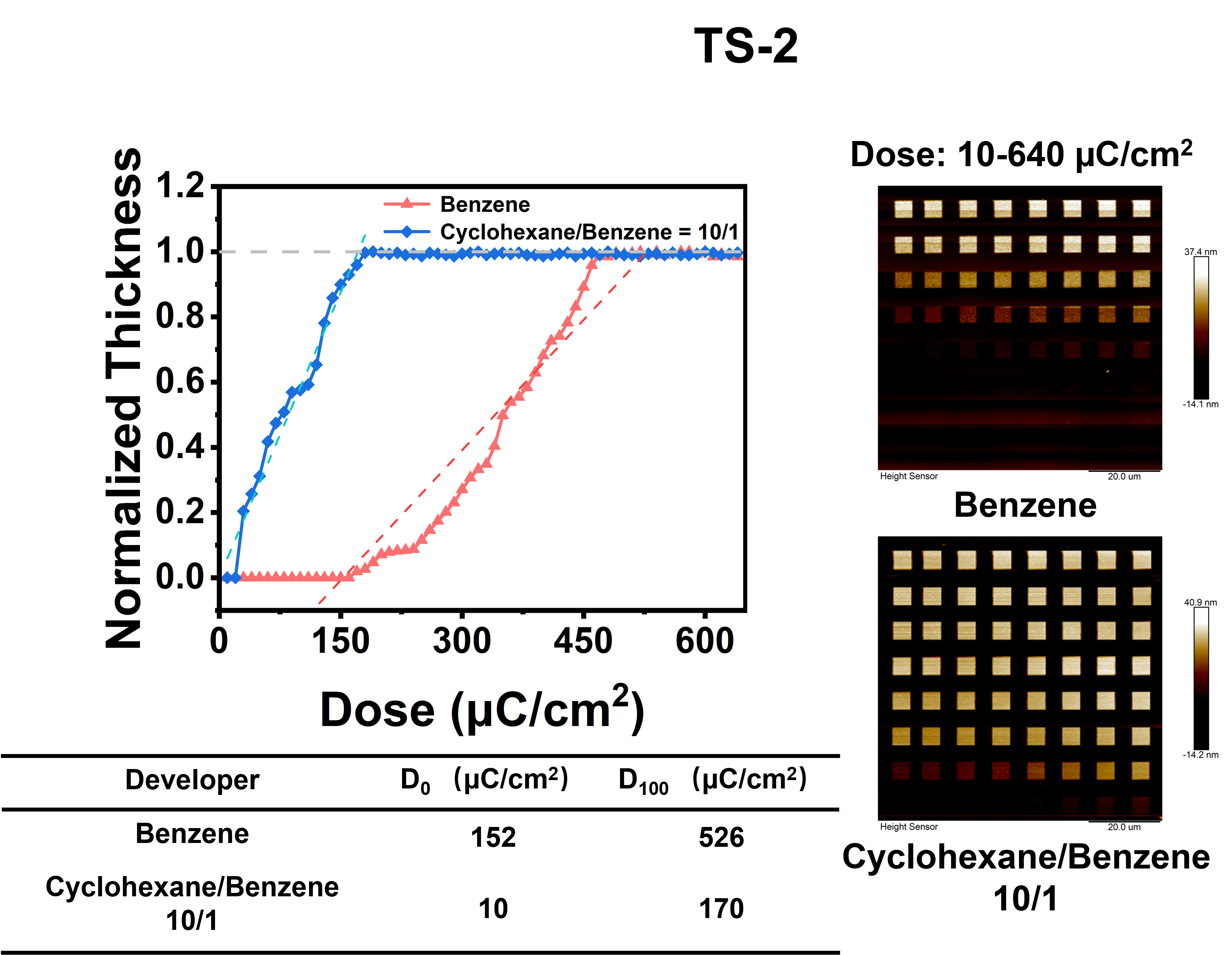


**Figure S30.** Dose array and contrast curve of **TS-2** before and after developer optimization.


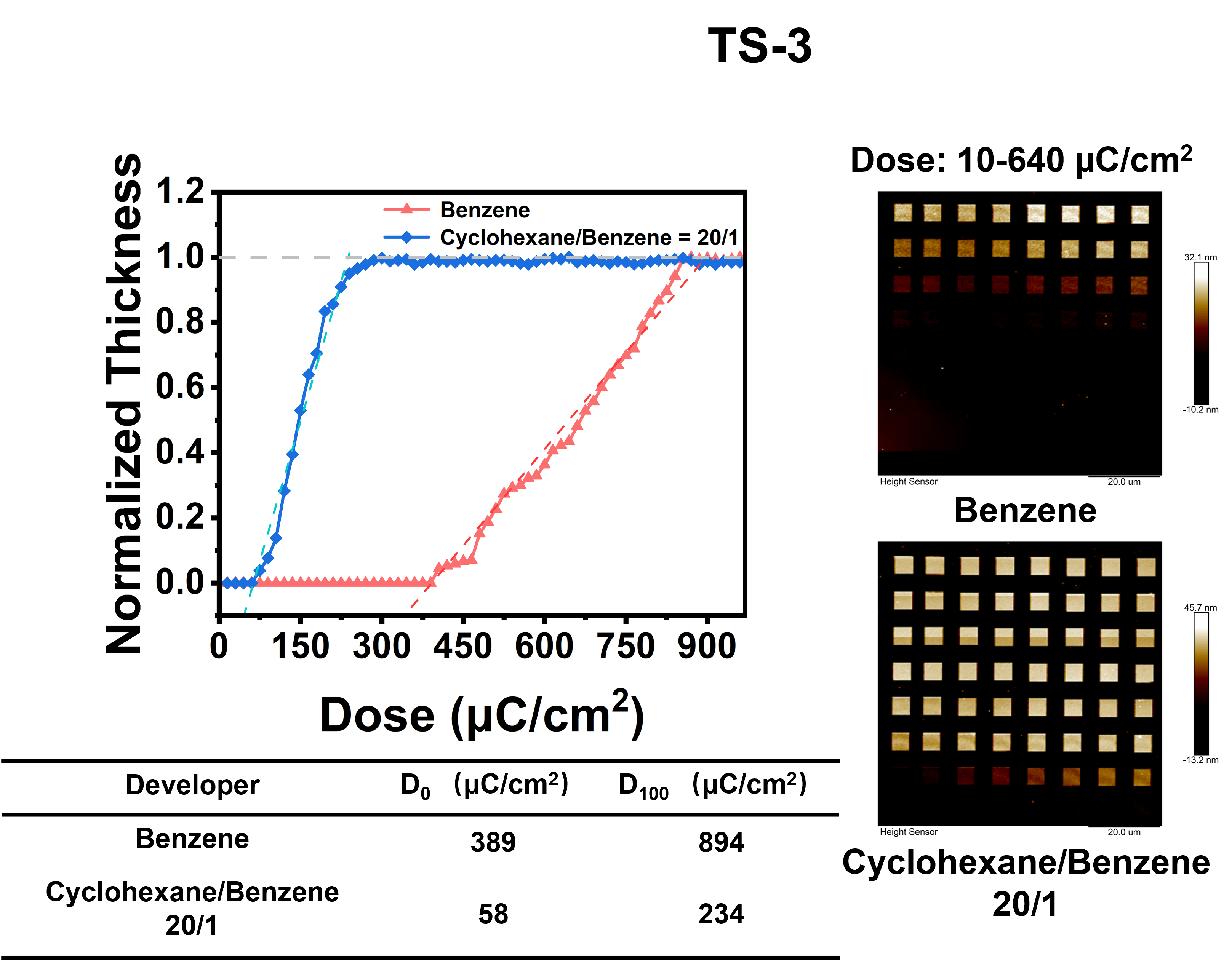


**Figure S31.** Dose array and contrast curve of **TS-3** before and after developer optimization.


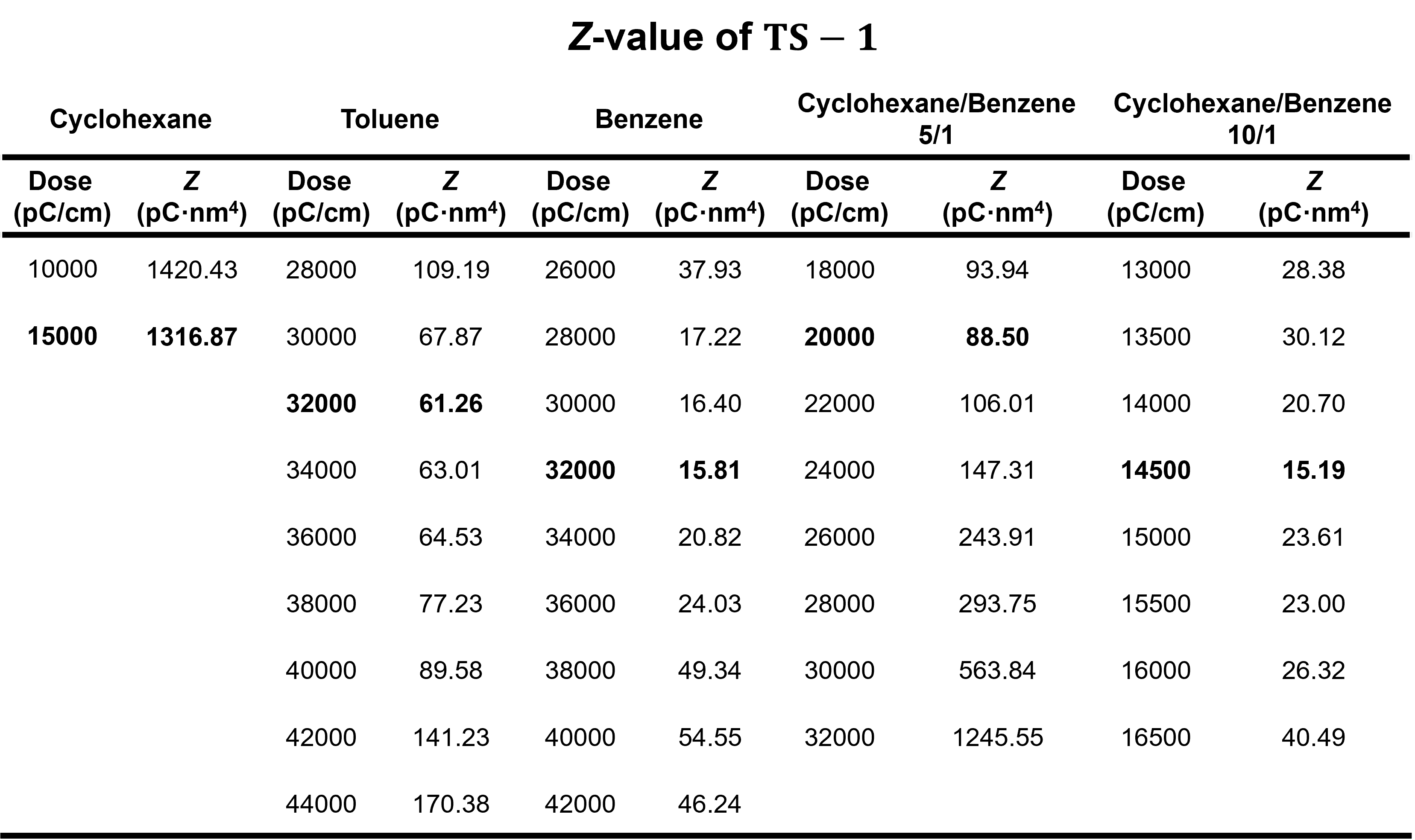


**Figure S32.** The *Z*-value of **TS-1** under different developer conditions.


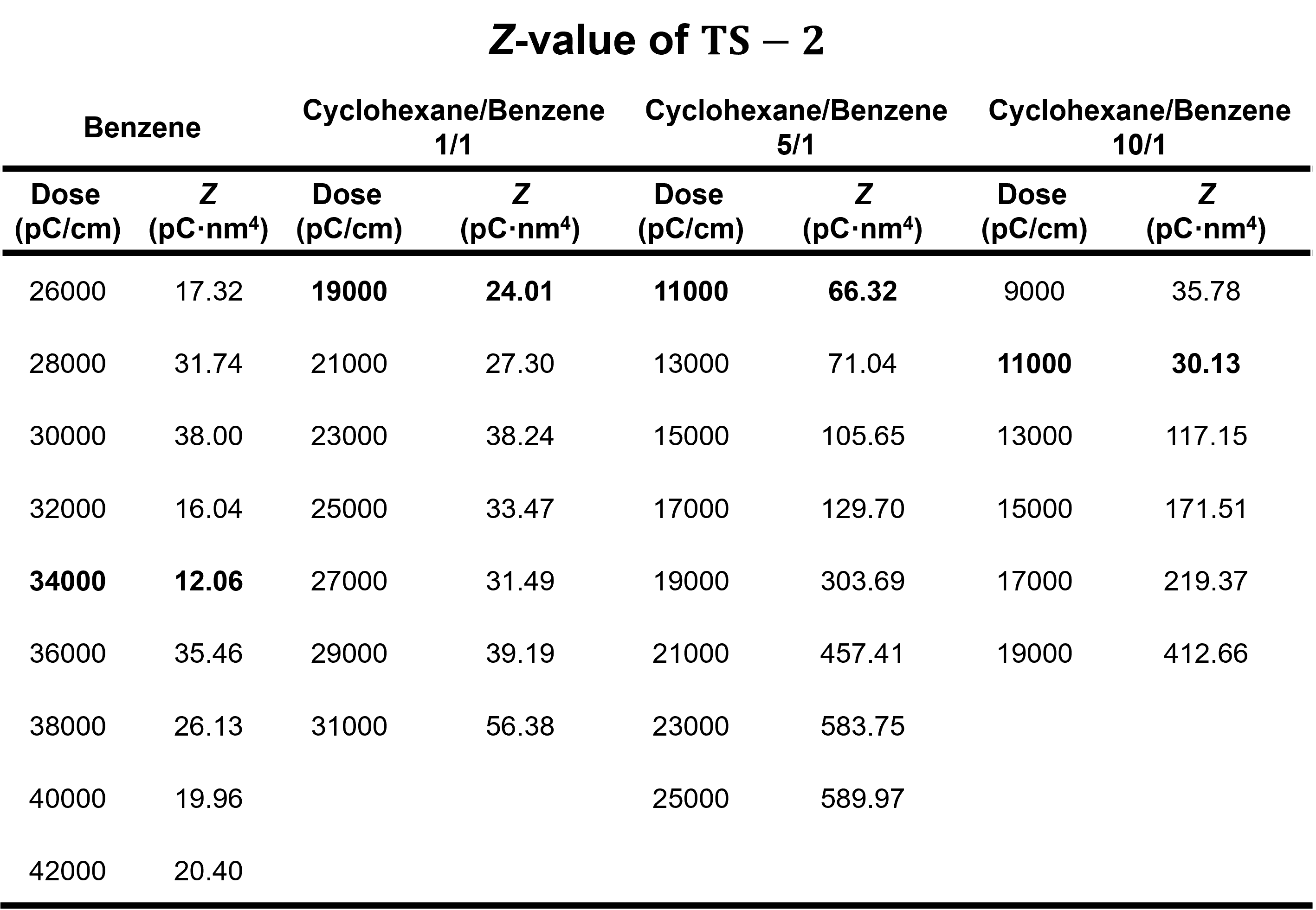


**Figure S33.** The *Z*-value of **TS-2** under different developer conditions.


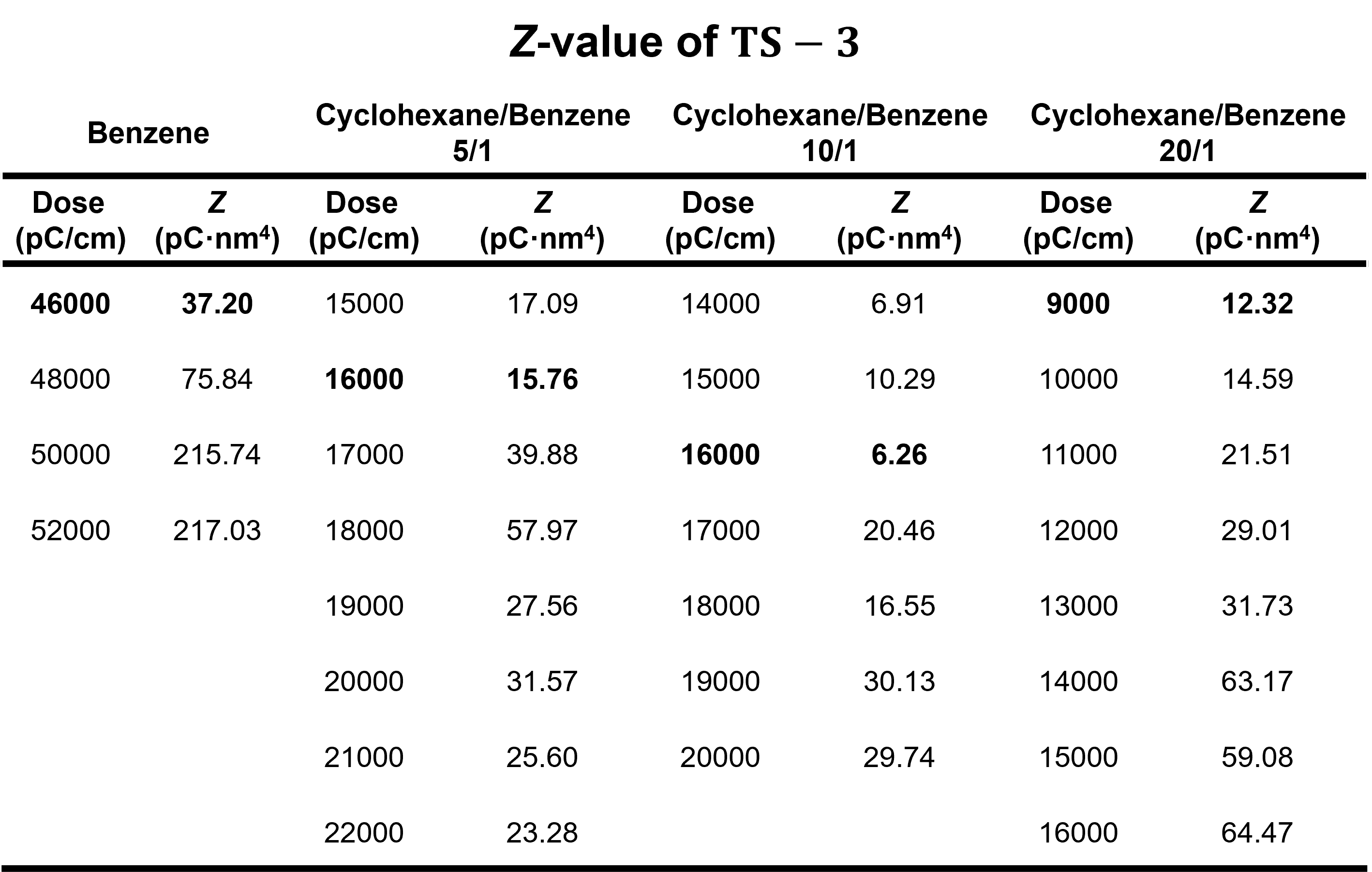


**Figure S34.** The *Z*-value of **TS-3** under different developer conditions.


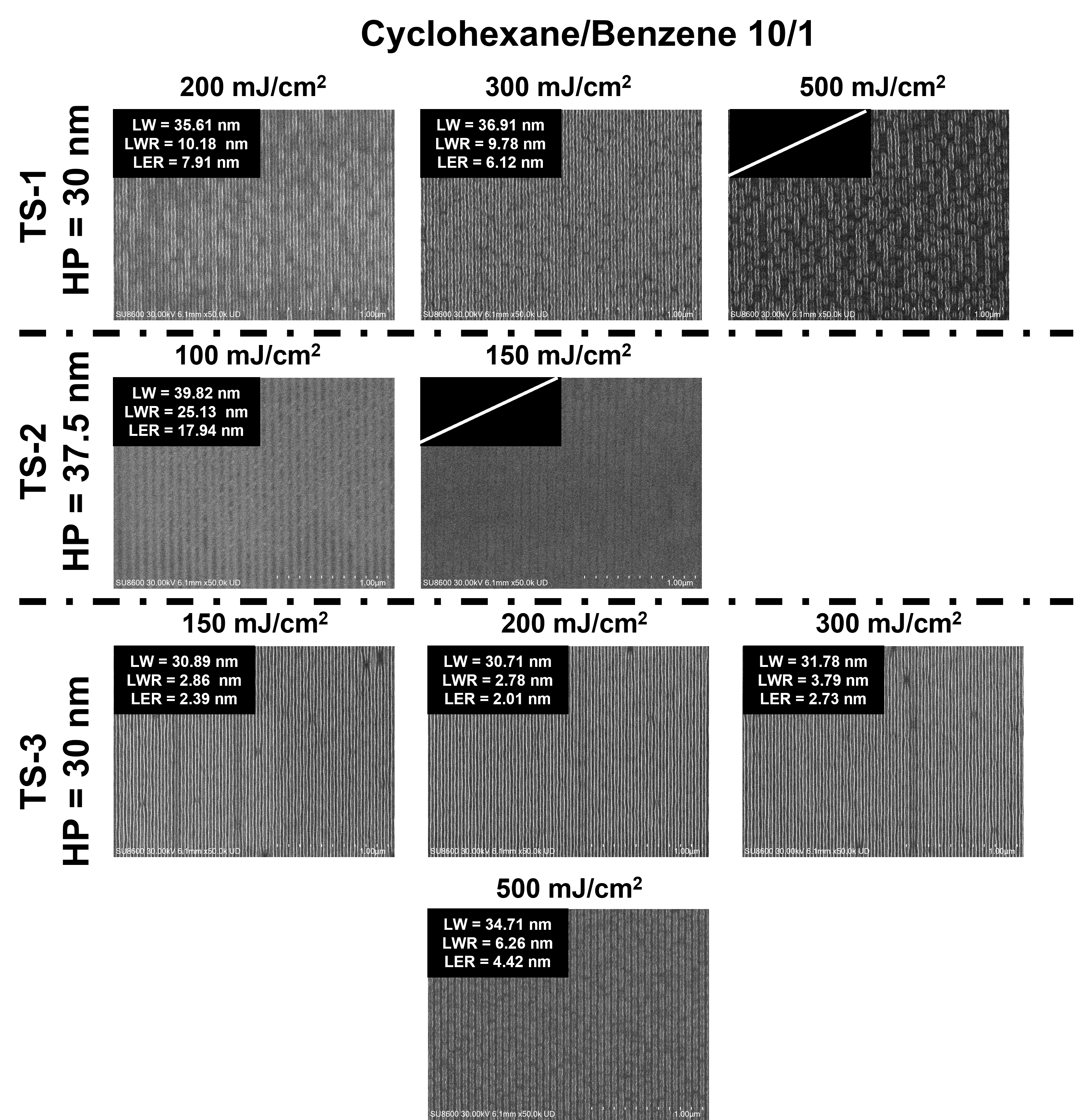


**Figure S35.** Patterning performance of **TS-1**, **TS-2** and **TS-3** under different EUV doses using cyclohexane/benzene = 10/1 as developer.


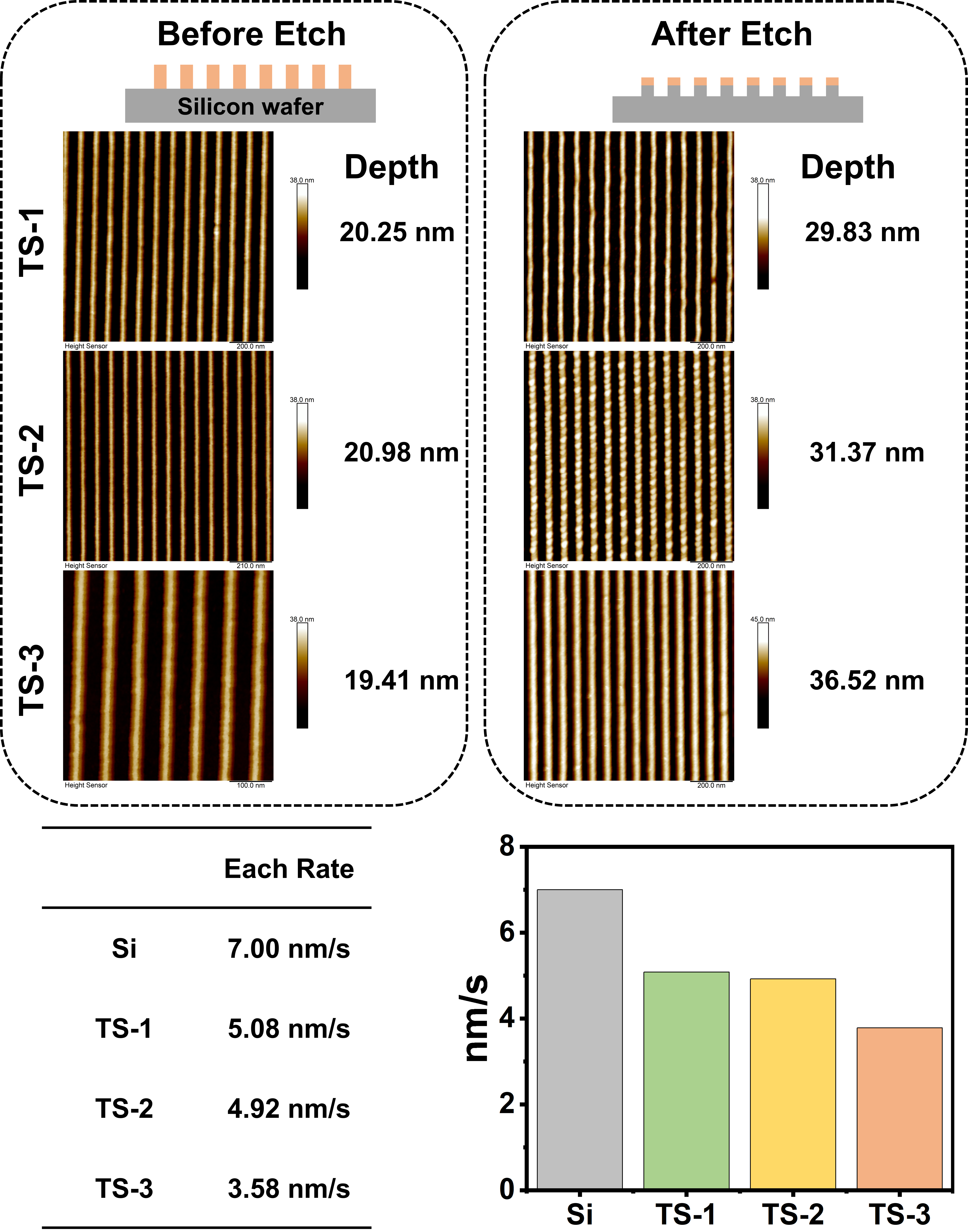


**Figure S36.** Thickness changes of **TS-1, TS-2**, and **TS-3** before and after etching for 5 s, and the etch rates of silicon wafers and the three clusters under these conditions

**Table S1.** Crystallographic data structure refinement for **TS-1**.

|  | TS-1 |
| --- | --- |
| Empirical formula | C_82_H_98_N_8_O_28_Sn_6_Ti_5_ |
| Formula weight | 2595.32 |
| Crystal system | monoclinic |
| Space group | C2/c |
| a/Å | 22.2507(4) |
| b/Å | 16.9475(3) |
| c/Å | 25.0480(5) |
| α/° | 90 |
| β/° | 97.144(2) |
| γ/° | 90 |
| Volume/Å^3^ | 9372.1(3) |
| Z | 4 |
| ρ_calc_ g/cm^3^ | 1.839 |
| μ/mm^-1^ | 2.050 |
| F(000) | 5120.0 |
| Crystal size/mm^3^ | 0.26 × 0.23 × 0.16 |
| Radiation | Mo Kα (λ = 0.71073) |
| 2Θ range for data collection/° | 3.69 to 52.746 |
| Index ranges | -27 ≤ h ≤ 27, -20 ≤ k ≤ 21, -31 ≤ l ≤ 31 |
| Reflections collected | 46764 |
| Independent reflections | 9591 [R_int_ = 0.0362, R_sigma_ = 0.0281] |
| Data/restraints/parameters | 9591/7/597 |
| Goodness-of-fit on F^2^ | 1.041 |
| Final R indexes [I>=2σ (I)] | R_1_ = 0.0282, wR_2_ = 0.0718 |
| Final R indexes [all data] | R_1_ = 0.0325, wR_2_ = 0.0735 |
| Largest diff. peak/hole / e Å^-3^ | 1.45/-0.84 |
| CCDC | 2471499 |

**Table S2.** Crystallographic data structure refinement for **TS-2**.

|  | TS-2 |
| --- | --- |
| Empirical formula | C_90_H_116_N_8_O_36_Sn_6_Ti_5_ |
| Formula weight | 2837.54 |
| Crystal system | triclinic |
| Space group | P-1 |
| a/Å | 13.20690(10) |
| b/Å | 30.1663(3) |
| c/Å | 30.9085(3) |
| α/° | 62.7910(10) |
| β/° | 87.0860(10) |
| γ/° | 88.4550(10) |
| Volume/Å^3^ | 10937.18(19) |
| Z | 4 |
| ρ_calc_ g/cm^3^ | 1.723 |
| μ/mm^-1^ | 1.770 |
| F(000) | 5640.0 |
| Crystal size/mm^3^ | 0.2 × 0.16 × 0.12 |
| Radiation | Mo Kα (λ = 0.71073) |
| 2Θ range for data collection/° | 3.446 to 52.744 |
| Index ranges | -16 ≤ h ≤ 16, -37 ≤ k ≤ 37, -38 ≤ l ≤ 38 |
| Reflections collected | 230801 |
| Independent reflections | 44754 [R_int_ = 0.0471, R_sigma_ = 0.0379] |
| Data/restraints/parameters | 44754/795/2950 |
| Goodness-of-fit on F^2^ | 1.052 |
| Final R indexes [I>=2σ (I)] | R_1_ = 0.0566, wR_2_ = 0.1256 |
| Final R indexes [all data] | R_1_ = 0.0851, wR_2_ = 0.1443 |
| Largest diff. peak/hole / e Å^-3^ | 1.45/-1.07 |
| CCDC | 2471496 |

**Table S3.** Crystallographic data structure refinement for **TS-3**.

|  | TS-3 |
| --- | --- |
| Empirical formula | C_72_H_78_N_8_O_30_Sn_2_Ti_4_ |
| Formula weight | 1964.40 |
| Crystal system | triclinic |
| Space group | P-1 |
| a/Å | 14.0572(3) |
| b/Å | 17.0389(4) |
| c/Å | 18.7336(4) |
| α/° | 85.535(2) |
| β/° | 72.503(2) |
| γ/° | 69.339(2) |
| Volume/Å^3^ | 4002.22(17) |
| Z | 2 |
| ρ_calc_ g/cm^3^ | 1.630 |
| μ/mm^-1^ | 1.083 |
| F(000) | 1988.0 |
| Crystal size/mm^3^ | 0.15 × 0.12 × 0.12 |
| Radiation | Mo Kα (λ = 0.71073) |
| 2Θ range for data collection/° | 4.474 to 52.744 |
| Index ranges | -17 ≤ h ≤ 17, -21 ≤ k ≤ 18, -22 ≤ l ≤ 23 |
| Reflections collected | 54635 |
| Independent reflections | 16334 [R_int_ = 0.0330, R_sigma_ = 0.0388] |
| Data/restraints/parameters | 16334/1669/1461 |
| Goodness-of-fit on F^2^ | 1.025 |
| Final R indexes [I>=2σ (I)] | R_1_ = 0.0845, wR_2_ = 0.2197 |
| Final R indexes [all data] | R_1_ = 0.1126, wR_2_ = 0.2409 |
| Largest diff. peak/hole / e Å^-3^ | 1.50/-1.03 |
| CCDC | 2471492 |

**Table S4.** Ligand dissociation temperature range.

| **Cluster** | **Bu** | **L1/L2** |
| --- | --- | --- |
| TS-1 | 282°C-388°C | 292°C-510°C |
| TS-2 | 271°C-380°C | 284°C-499°C |
| TS-3 | 258°C-386°C | 364°C-396°C |

**Table S5.** DIP-MS results of **TS-1**, **TS-2**, and **TS-3**.

|  | **TS-1** | | **TS-2** | | **TS-3** | | |
| --- | --- | --- | --- | --- | --- | --- | --- |
| **Ligand** | **m/z** | **Fragment Ion** | **m/z** | **Fragment Ion** | **m/z** | **Fragment Ion** |  |
| L1/2 | 119.0367 | [C_7_H_5_NO]^+ 2·^ | 149.0475 | [C_8_H_7_NO_2_]^+ 2·^ | 149.0471 | [C_8_H_7_NO_2_]^+ 2·^ |  |
|  | 107.0492 | [C_7_H_7_O]^+ ·^ | 119.0367 | [C_7_H_5_NO]^+ 2·^ | 119.0366 | [C_7_H_5_NO]^+ 2·^ |  |
|  | 94.0414 | [C_6_H_6_O]^+^ | 91.0543 | [C_7_H_7_]^+ ·^ |  |  |  |
|  | 77.0386 | [C_6_H_5_]^+ ·^ | 77.0386 | [C_6_H_5_]^+ ·^ |  |  |  |
| *n*Bu | 57.0699 | [C_4_H_9_]^+ ·^ | 57.0699 | [C_4_H_9_]^+ ·^ | 55.0543 | [C_4_H_7_]^+ 3·^ |  |
|  | 56.0621 | [C_4_H_8_]^+ 2·^ | 55.0543 | [C_4_H_7_]^+ 3·^ | 50.0151 | [C_4_H_2_]^+ 8·^ |  |
|  | 55.0542 | [C_4_H_7_]^+ 3·^ | 51.0299 | [C_4_H_3_]^+ 7·^ | 41.0384 | [C_3_H_5_]^+ 3·^ |  |
|  | 54.0464 | [C_4_H_6_]^+ 4·^ | 41.0385 | [C_3_H_5_]^+ 3·^ | 39.0228 | [C_3_H_3_]^+ 5·^ |  |
|  | 51.0299 | [C_4_H_3_]^+ 7·^ | 39.0229 | [C_3_H_3_]^+ 5·^ |  |  |  |
|  | 50.0151 | [C_4_H_2_]^+ 8·^ |  |  |  |  |  |
|  | 49.0073 | [C_4_H]^+ 9·^ |  |  |  |  |  |
|  | 48.5892 | [C_4_]^+ 10·^ |  |  |  |  |  |
|  | 39.0229 | [C_3_H_3_]^+ 5·^ |  |  |  |  |  |

**Table S6.** Specific process parameters for the ICP etching methods.

| **Substrate** | **Time** | **Pressure** | **Gas/sccm** | **RF Power** |
| --- | --- | --- | --- | --- |
| Silicon | 4 s | 4.13E^-2^ Torr | **SF_6_** | 200W |
|  |  |  | 30 |  |

# References

[1] J. Wang, M. Luo, Q. Lin, *Journal of Solid State Chemistry* **2024**, 335, 124723.

[2] a) O. V. Dolomanov, L. J. Bourhis, R. J. Gildea, J. A. K. Howard, H. Puschmann, *Journal of Applied Crystallography* **2009**, 42, 339; b) D. Leggas, O. V. Tsodikov, *Acta Crystallographica Section A Foundations and Advances* **2015**, 71, 319.

[3] a) T. Lu, F. Chen, *Journal of Molecular Graphics and Modelling* **2012**, 38, 314; b) J. Zhang, T. Lu, *Physical Chemistry Chemical Physics* **2021**, 23, 20323.
